# Catalpol attenuates hepatic glucose metabolism disorder and oxidative stress in triptolide-induced liver injury by regulating the SIRT1/HIF-1α pathway

**DOI:** 10.7150/ijbs.97362

**Published:** 2024-08-01

**Authors:** Weijue Nie, Hong Zhu, Xin Sun, Jie Zhou, Heng Xu, Zhichao Yu, Minghao Lu, Baoping Jiang, Lingling Zhou, Xueping Zhou

**Affiliations:** 1 The First Clinical Medical College, Nanjing University of Chinese Medicine, Nanjing 210023, China.; 2 School of Chinese Medicine, Nanjing University of Chinese Medicine, Nanjing 210023, China.; 3 Department of Pharmacology, School of Pharmacy, Nanjing University of Chinese Medicine, Nanjing 210023, China.

**Keywords:** Catalpol, Drug-induced liver injury, Oxidative stress, Energy metabolism, Hypoglycemia

## Abstract

Triptolide (TP), known for its effectiveness in treating various rheumatoid diseases, is also associated with significant hepatotoxicity risks. This study explored Catalpol (CAT), an iridoid glycoside with antioxidative and anti-inflammatory effects, as a potential defense against TP-induced liver damage. *In vivo* and *in vitro* models of liver injury were established using TP in combination with different concentrations of CAT. Metabolomics analyses were conducted to assess energy metabolism in mouse livers. Additionally, a Seahorse XF Analyzer was employed to measure glycolysis rate, mitochondrial respiratory functionality, and real-time ATP generation rate in AML12 cells. The study also examined the expression of proteins related to glycogenolysis and gluconeogenesis. Using both *in vitro SIRT1* knockout/overexpression and* in vivo* liver-specific *SIRT1* knockout models, we confirmed SIRT1 as a mechanism of action for CAT. Our findings revealed that CAT could alleviate TP-induced liver injury by activating SIRT1, which inhibited lysine acetylation of hypoxia-inducible factor-1α (HIF-1α), thereby restoring the balance between glycolysis and oxidative phosphorylation. This action improved mitochondrial dysfunction and reduced glucose metabolism disorder and oxidative stress caused by TP. Taken together, these insights unveil a hitherto undocumented mechanism by which CAT ameliorates TP-induced liver injury, positioning it as a potential therapeutic agent for managing TP-induced hepatotoxicity.

## Introduction

Triptolide (TP), a potent compound derived from the traditional Chinese medicinal plant *Tripterygium wilfordii* Hook f. (TwHf), has demonstrated effectiveness in treating various autoimmune disorders, including rheumatoid arthritis, nephritis, and systemic lupus erythematosus 1-3. Notably, TP is considered a promising candidate for translation from traditional to modern medicine 4. However, growing clinical evidence highlights significant adverse reactions associated with TP, particularly its hepatotoxic effects, which have garnered considerable attention 5.

The mechanisms underlying TP-induced hepatotoxicity are complex and involve oxidative stress, dysregulation of drug-metabolizing enzymes, and an imbalance in Th17/Treg cells 6-8. These mechanisms are not yet fully understood. Studies have shown a strong link between TP and mitochondrial dysfunction and disturbances in energy metabolism. TP appears to cause mitochondrial oxidative stress, leading to a decrease in mitochondrial membrane potential and enzyme activities. This impairment compromises the mitochondrial respiratory chain, disrupting energy production and distribution 9. Additionally, the decline in mitochondrial function alters fatty acid and glucose metabolism, and impairs mitochondrial oxidative phosphorylation (OXPHOS). This increases the production of reactive oxygen species (ROS), exacerbating cellular and tissue damage and further impacting energy metabolism 10.

Sirtuin 1 (SIRT1), a class III protein deacetylase encoded by the *SIRT1* gene, relies on NAD^+^ for its enzymatic activity. It plays a crucial role in various cellular metabolic processes, such as regulating the cell cycle, maintaining mitochondrial homeostasis, and controlling glucose and lipid metabolism 11. SIRT1 helps prevent various types of liver damage by deacetylating certain transcription control factors, thereby influencing hepatic energy metabolism (e.g., gluconeogenesis, glycogenolysis, and fatty acid oxidation), controlling oxidative stress, and suppressing NF-κB signaling, a key pathway in liver inflammation 12. The hypoxia-inducible factor-1 (HIF-1) is a transcription factor complex composed of an oxygen-regulated HIF-1α subunit and a constant HIF-1β subunit. HIF-1 functionality depends on the degradation of HIF-1α subunits, which stimulate the transcription of numerous genes, particularly those involved in metabolic pathways such as glycolysis, OXPHOS, and nucleotide metabolism 13. Studies suggest that HIF-1α plays a significant role in the onset and progression of several liver diseases, including non-alcoholic fatty liver disease (NAFLD), liver fibrosis, and drug-induced liver injury (DILI) 14. Convincing evidence proposes that SIRT1 can inhibit glycolysis by deactivating HIF-1α, and its function is meticulously regulated by NAD^+^ levels, a metabolite of mitochondrial OXPHOS 15.

Catalpol (CAT), an iridoid glycoside found in several plants, including *Rehmannia glutinosa* (Gaertn.) DC., exhibits antioxidant, anti-inflammatory, and anti-apoptotic characteristics 16. Previous studies have demonstrated that CAT ameliorates TP-induced liver injury by suppressing endoplasmic reticulum stress, inducing Cytochrome P450 enzyme activity, and regulating lipid metabolism 17-19. Furthermore, CAT protects against adriamycin-induced kidney damage by activating SIRT1 to reduce TRPC6 and increase MRP2 expression 20. Moreover, CAT enhances mitochondrial biogenesis, leading to the amelioration of mitochondrial damage in skeletal muscle 21, suggesting its potential role in regulating energy metabolism.

In this study, we assessed the impact of CAT in ameliorating TP-induced hepatic damage in C57BL/6J mice and explored its underlying mechanisms. Our findings demonstrated that CAT treatment improved TP-induced hepatic glucose metabolism disorder and oxidative stress by modulating the SIRT1/HIF-1α pathway. This modulation is associated with restoring the balance between glycolysis and OXPHOS and improving mitochondrial dysfunction. These results suggest that CAT might be a promising therapeutic strategy for TP-induced liver injury.

## Materials and Methods

### Cell culture and treatment

AML12 cells, an immortalized standard mouse hepatocyte cell line, were purchased from the Chinese Academy of Science Committee Type Culture Collection Cell Bank (SCSP_550) and cultured in DMEM/F12 media (Gibco) containing 10% fetal bovine serum, ITS supplement (Beyotime), 40 ng/ml dexamethasone, and 1% Penicillin-Streptomycin (Invitrogen) under controlled conditions (37°C, 5% CO_2_). Both TP and CAT were dissolved in DMSO and diluted in a complete medium, ensuring the DMSO content remained below 0.1% (v/v) in all experiments. To determine the optimal concentration for inducing hepatic injury, AML12 cells were treated with varying TP concentrations (0-1920 nM) for 12-48 h. Subsequently, 60 nM TP for 36 h was selected for further experiments. To assess the cytoprotective effects of CAT, cells were pre-treated with various CAT concentrations (100-400 nM) for 6 h before co-incubation with 60 nM TP for 36 h. Following this protocol, cells were divided into five groups: (1) Control group (0.1% DMSO only); (2) TP group (60 nM TP, 36 h); (3) TP + CAT100 group (100 nM CAT pre-treatment, followed by 60 nM TP co-incubation); (4) TP + CAT200 group (200 nM CAT pre-treatment, followed by 60 nM TP co-incubation); and (5) TP + CAT400 group (400 nM CAT pre-treatment, followed by 60 nM TP co-incubation).

### Lentivirus packaging and transduction

The cDNA encoding mouse SIRT1 (NM_019812.3) was inserted into the CMV promoter-dependent lentivirus vector pLenti-GIII-CMV-CBH-GFP-2A-Puro (Applied Biological Materials Inc., Zhenjiang, China), which expresses both the target gene and enhanced green fluorescent protein (eGFP). To knock out *SIRT1* in cells, the U6 promoter-dependent lentivirus vector pLenti-U6-sgRNA-SFFV-Cas9-2A Puro (Applied Biological Materials Inc., Zhenjiang, China) was used. The target sequence for *SIRT1* was 5′-TCCGCTTTGGTGGTTCTGAA-3′. All the engineered lentiviral vectors were then packaged into 293T cells to produce a lentivirus that either overexpresses *SIRT1* or knocks it out. An empty vector served as a control.

For lentivirus infection, 5 × 10⁵ AML12 cells were seeded per well in 6-well plates and incubated for 24 hours. The cells were then infected with *SIRT1* overexpressing lentivirus, *SIRT1* knockout lentivirus, or control lentivirus, all in the presence of 8 μg/ml polybrene. After 12 hours, the culture medium was replaced with a fresh DMEM/F12 complete medium for another 12 hours. Stable cell lines were selected using puromycin (2 μg/ml). Transduction efficiency (GFP expression level) for the overexpressing line was evaluated by fluorescence microscopy. SIRT1 protein knockdown in the knockout cell line was further evaluated by western blot. AML12 cells infected with an empty lentiviral vector served as a control.

### CCK8 assay

AML12 cells were seeded at a density of 9,000 cells/well (100 μl medium per well) in 96-well plates with five replicates per condition. Following seeding, the cells were exposed to various concentrations of CAT and TP before incubating for 36 h. Cell viability was subsequently determined using a CCK8 assay, following the supplier's protocol.

### Animal experiments and design

All animal care and experimental procedures were conducted in accordance with the guidelines of China's National Institutes of Health and were approved by the Animal Care and Use Committee of Nanjing University of Chinese Medicine. Animals were housed in accordance with standard guidelines with enrichment to promote well-being. The animal research performed complied with the ARRIVE guidelines 2.0.

Female C57BL/6J mice, aged around 18 grams (± 0.5 g), were obtained from Beijing Vital River Laboratory Animal Technology Co., Ltd. (Beijing, China). These mice were housed under controlled conditions with consistent temperature, humidity, and lighting. They had unrestricted access to both food and water throughout the experiment. Following the methods established in previous research 22, the mice were randomly divided into two main groups: normal molding and AAV intervention + modeling.

1. The normal molding group consisted of three subgroups (n = 6 each):

(1) Control group (*n* = 6): Mice received a daily gavage of 0.5% sodium carboxymethyl cellulose solution for two weeks after a one-week acclimation period.

(2) TP group (*n* = 6): Mice received a daily gavage of 0.6 mg/kg triptolide for two weeks after a one-week acclimation period.

(3) TP + CAT group (*n* = 6): Mice received a daily gavage of 0.6 mg/kg triptolide combined with varying doses (1.5, 3, and 4.5 mg/kg) of catalpol for two weeks after a one-week acclimation period. Catalpol administration began three days prior to triptolide.

2. The AAV intervention + modeling group consisted of four subgroups (n = 6 each):

1). AAV-Scr + TP group (*n* = 6): Mice received injections of AAV-Scr once a week for two weeks, followed by a two-week acclimation period and then a daily gavage of 0.6 mg/kg triptolide for two weeks.

2). AAV-Scr + TP + CAT group (*n* = 6): Mice received injections of AAV-Scr once a week for two weeks, followed by a two-week acclimation period, pre-administration of 4.5 mg/kg catalpol for three days, and then a daily gavage of 0.6 mg/kg triptolide and 4.5 mg/kg catalpol for two weeks.

3). AAV-SIRT1 + TP group (*n* = 6): Mice received injections of AAV-SIRT1 once a week for two weeks, followed by a two-week acclimation period and then a daily gavage of 0.6 mg/kg triptolide for two weeks.

4). AAV-SIRT1 + TP + CAT group (*n* = 6): Mice received injections of AAV-SIRT1 once a week for two weeks, followed by a two-week acclimation period, pre-administration of 4.5 mg/kg catalpol for three days, and then a daily gavage of 0.6 mg/kg triptolide and 4.5 mg/kg catalpol for two weeks.

Triptolide and catalpol were dissolved in a 0.5% sodium carboxymethyl cellulose solution.

Body weight measurements were recorded every two days throughout the experiment. After a two-week administration period, the mice were euthanized. Serum and liver tissues were then collected for subsequent analyses. Blinding was employed during these analyses.

### Adeno-associated virus packaging and infection

Targeting sequences within exon three of murine *SIRT1* were selected and cloned into an AAV-CRISPR-Cas9 vector with self-cleaving spacers (pAAV-PGK-saCas9-U6-sgRNAsa, Applied Biological Materials Inc., Zhenjiang, China). The engineered AAV vector contains the SaCas9 enzyme and a U6 promoter-driven sgRNA (sgRNAa) targeting *SIRT1*. The sgRNA sequence was: 5'-CCGUCUCUGUGUCACAAAUTT-3'. This vector or a control vector containing a scrambled sgRNA was packaged in HEK293T cells using standard transfection protocols. The AAV vector used was liver-specific serotype 8. Mice were injected in the tail vein with a dose of 2 × 10^11^ viral genomes per mouse. Mice injected with the control AAV vector (AAV-Scr) served as a control.

### Histopathology analysis

Liver tissues were fixed in a 4% neutral formaldehyde solution, followed by paraffin embedding and sectioning into 4-5 μm thick sections. Routine Hematoxylin and Eosin (H&E) staining was performed on the sections, and pathological changes in the hepatic tissues were observed using an optical microscope (DM4 B, Leica, Hamburg, Germany). To assess glycogen content in liver tissues and AML12 cells, Periodic Acid Schiff (PAS) staining was performed.

### Immunofluorescence analysis

AML12 cells were seeded in 24-well plates and treated with TP and CAT for 36 h. Subsequently, the cells were washed with chilled PBS and fixed in a 4% neutral formaldehyde solution at room temperature. Following fixation, the cells underwent three PBS washes and were then blocked with 1% BSA at ambient temperature for 1 hour. Three additional washes were performed before incubation with primary and secondary antibodies (detailed in Table S1) in a mixture of PBS, 0.3% (v/v) Triton X-100, and 1% BSA. The cell nuclei were then stained with DAPI for visualization, followed by the application of an anti-fluorescence quenching sealant. Examination of the slides was carried out using a confocal laser-scanning microscope (TCS SP8, Leica, Hamburg, Germany). This methodology was also applied to hepatic frozen sections.

### Measurement of the mitochondrial membrane potential (ΔΨm)

Changes in ΔΨm were evaluated using a ΔΨm assay kit with JC-1 (HY-15534, MCE, Shanghai, China), following the manufacturer's guidelines. After treatment, cells from each group were harvested, washed once with PBS, and resuspended in a complete culture medium or assay buffer at a defined cell density. JC-1 was added to the cell suspension to reach a final concentration of 2 μM. After incubating under dark conditions at 37 °C for 15-20 minutes, the cells were rinsed twice with PBS and then re-suspended in 500 μl of PBS. Flow cytometry was used for measurement, followed by an analysis of the fluorescence intensity.

### Quantitative real-time PCR for the mtDNA content

Genomic DNA was isolated from cryopreserved liver specimens using a tissue DNA extraction kit (Servicebio, Wuhan, China). The concentration of the isolated DNA was then quantified using a DS-11 FX+ spectrophotometer (DeNovix). Primers specific for mouse cytochrome β and β-actin genes (sequences listed in Table S2) were obtained from Sangon Biotechnology (Shanghai, China). The Hieff qPCR SYBR Green Master Mix (Yeasen, Shanghai, China) was used for amplification and quantification of mtDNA.

### Biochemical analysis

After overnight storage at 4 °C, sera were isolated from blood samples by centrifugation at 3000 × g for 15 min and stored at -80°C. Supernatants from cell cultures were collected for further analyses. The levels of aspartate aminotransferase (AST), alanine aminotransferase (ALT), and lactate dehydrogenase (LDH) were quantified using commercially available kits (Jiancheng Co., Nanjing, China). Levels of MDA, GSH/GSSG, and NAD^+^/NADH in liver tissues or AML12 cells were determined using Lipid Peroxidation MDA Assay Kit, GSH/GSSG Assay Kit, and NAD^+^/NADH Assay Kit, respectively (all obtained from Beyotime, Shanghai, China). Glycogen content in the liver and AML12 cells was measured using a glycogen assay kit (Solarbio, Beijing, China). Blood glucose was measured from the tail vein using a glucose monitoring system (Roche, Germany). Serum insulin and glucagon concentrations, along with cAMP levels in the liver, were assessed using their respective ELISA kits (AiFang Bio, Hunan, China).

### Real-time quantitative PCR

Total RNA was extracted from frozen tissue specimens using Trizol (Invitrogen, Carlsbad, CA, USA) following the manufacturer's instructions. Double-stranded cDNA synthesis was then carried out from 1 μg of total RNA using Hifair III 1st Strand cDNA Synthesis SuperMix (Yeasen, Shanghai, China). Real-time PCR was performed with Hieff qPCR SYBR Green Master Mix (Yeasen, Shanghai, China) and primers listed in Supplementary Table S2. All samples were run in triplicate and subjected to 40 cycles of amplification on a Roche Applied Science LightCycler 96 Instrument (Roche, Basel, Switzerland). β-actin served as the internal control gene for each sample, and relative gene expression was determined using the 2^-ΔΔCT^ methodology.

### Western blotting

Cellular or preserved liver proteins were harvested. A total of 40 μg protein was loaded onto each lane of 10% and 12.5% SDS-polyacrylamide gels. Following electrophoresis, proteins were transferred to PVDF membranes. The membranes were incubated overnight at 4 °C with primary antibodies listed in Supplementary Table S1. After washing, they were incubated with a secondary antibody diluted 1:10,000. Protein signals were detected using an ECL plus chemiluminescence kit (Millipore) and visualized with the Image Analyzer ChemiDoc XRS (Bio-Rad). Changes in protein expression were quantified using Image J software with β-actin as an internal control.

### Co-immunoprecipitation

Cells were cultured in 10-cm dishes and then treated with TP, TP + CAT, or left untreated for 36 h. 1mg of total protein from each sample was incubated overnight at 4 °C with either an anti-SIRT1 antibody or an anti-HIF-1α antibody. Immunoprecipitation was then performed using 30 µl of Protein A/G Magnetic Beads (MCE, Shanghai, China) for 4 h at 4 °C. The immunoprecipitated complexes were subsequently immunoblotted using antibodies against SIRT1, HIF-1α, or acetyl-lysine.

### Analyzing glucose metabolism phenotype with Seahorse analyzer

AML12 cells were seeded at a density of 3 × 10^4^ cells per well in Seahorse XF 24-well culture plates containing growth medium and allowed to adhere overnight. The wells were then treated with TP, or TP + CAT (100, 200, and 400nM CAT, respectively) for 36 h. Following treatment, the cell cultures were incubated for 1 hour at 37 °C in a CO_2_-free environment. Assays assessing cellular glycolytic rate, mitochondrial stress, and real-time ATP production were performed using a Seahorse XF Analyzer (Seahorse Agilent, USA). The protocol for determining oxygen consumption rates (OCR) and extracellular acidification rates (ECAR) associated with glycolysis was followed. Data analysis was performed using Seahorse Wave software, version 2.6.3.

#### Glycolytic rate assay

The glycolytic rate assay allows for precise measurement of extracellular acidification solely attributed to glycolysis by excluding mitochondrial contributions. A significant correlation was observed between the real-time measured glycolytic proton efflux rate (glycoPER) via ECAR and OCR analysis, and the rate of extracellular lactate production. Following the standard protocol, 3 × 10^4^ cells were cultured per well and treated with either TP or TP + CAT for 36 hours. This approach enabled accurate assessment of basal glycolytic rates and compensatory glycolysis following Rot/AA injection (0.5 µM). Subsequent administration of 2-deoxy-D-glucose (2-DG, 50 mM) inhibited glycolytic acidification, resulting in declined Proton Efflux Rate (PER). This confirmed that the previously observed PER was predominantly due to glycolysis.

#### Cell mitochondrial stress assay

Basal respiration, ATP-linked respiration, and reserve respiratory capacity were evaluated in AML12 cells treated with TP or TP + CAT using the Seahorse Mito Stress Test protocol. Basal oxygen consumption was measured after injection of glutamine/pyruvate (2 and 1 mM, respectively). Inhibition of mitochondrial ATP production was achieved through oligomycin (1.5 µM) injection, allowing the determination of mitochondrial oxidative leakage. Maximum uncoupling of the mitochondrial electron transport chain was achieved by FCCP injection (1 µM). Subsequently, rotenone/antimycin A (Rot/AA, 0.5 µM), inhibitors of complex I and III, were introduced to estimate non-mitochondrial oxygen consumption. To evaluate basal respiration, oxidative leakage, and maximal respiration, the value of non-mitochondrial respiration was subtracted.

#### Real-time ATP rate assay

OCR and ECAR assays are used to assess the rates of mitochondrial ATP (mitoATP) and glycolytic ATP (glycoATP) production using established algorithms. We investigated the real-time rates of ATP synthesis from both the tricarboxylic acid (TCA) cycle and glycolysis following TP or TP + CAT treatment. 3 × 10^4^ cells/well were seeded in culture plates and treated with TP or TP + CAT for 36 hours. Concurrently, the rates of proton (H^+^) efflux and oxygen (O_2_) consumption were quantified. Using data obtained under basal conditions and following the sequential addition of mitochondrial inhibitors (1.5 µM oligomycin and 0.5 µM Rot/AA), we calculated total cellular ATP production rates and further distinguished pathway-specific mitoATP and glycoATP production rates.

### Targeted metabolomic analysis

This study identified a total of 32 key metabolites associated with the glycolytic pathway, TCA cycle, OXPHOS, and their respective cofactors using ultra-performance liquid chromatography-tandem mass spectrometry (UPLC-MS/MS).

#### Liver metabolomic sample preparation

A 50 mg frozen liver sample was combined with 200 μl of pre-cooled distilled water, followed by 800 μl of chilled methanol:acetonitrile (1:1 v/v). The mixture was then sonicated for 1 hour and incubated at -20 °C for 1 hour. Following centrifugation at 16,000 rpm for 20 minutes at 4 °C, an internal standard of Succinate-d6 was added to the supernatant. The supernatant was then freeze-dried and stored at -80 °C. Finally, the lyophilized residue was resuspended in 100 μl of acetonitrile solution (1:1 v/v) before UPLC-MS/MS analysis.

#### UPLC-MS/MS global profiling of liver metabolites

The UPLC-MS/MS analysis was conducted using a 1290 Infinity UPLC system (Agilent, California, USA) coupled with a 5500 QTRAP mass spectrometer (AB SCIEX, Toronto, Canada). Sample separation was achieved using an ACQUITY UPLC BEH Amide column (2.1 mm × 100 mm, 1.7 μm). The column temperature was maintained at 40°C, and the flow rate was set at 300 μl/min (Waters, Milford, USA). The mobile phase consisted of a linear gradient of (A) 5% aqueous acetonitrile (v/v) and (B) 95% aqueous acetonitrile (v/v), each containing 10 mM ammonium acetate. The gradient elution program was as follows: 0-2 min (95% B); 2-9 min (95%-70% B); 9-10 min (70%-30% B); 10-11 min (30% B); 11-11.5 min (30%-95% B); 11.5-15 min (95% B). The mass spectrometer parameters were set with the ion spray voltage at 5.5 kV (positive) and 4.5 kV (negative). The curtain gas, nebulizer gas, and auxiliary gas were maintained at 35, 40, and 50 psi, respectively, with the source temperature controlled at 550°C.

#### Data processing and analysis

Data from the UPLC-MS/MS analysis was processed with MultiQuant software 3.0.2 (AB SCIEX, Toronto, Canada) for initial analysis, including peak detection, identification, and integration. This detection and alignment process was conducted on all samples by referring to their corresponding internal standards. A multivariate statistical evaluation, specifically, using Principal Component Analysis (PCA), was performed using Metaboanalyst 6.0 (http://www.metaboanalyst.ca/). A t-test was employed to confirm all differentially expressed energy metabolites between the two groups, with a significance threshold set at a p-value of 0.05.

### PTT and IPGTT tests

Following two weeks of daily oral administration of either 0.6 mg/kg TP or 4.5 mg/kg CAT, female mice underwent pyruvate tolerance tests (PTT) and intraperitoneal glucose tolerance tests (IPGTT). After a 16-hour fast, the mice received an intraperitoneal injection of sodium pyruvate (Sigma) at a dose of 1.5 g/kg body weight for the PTT or D-glucose (Sigma) at 1 g/kg body weight for the IPGTT. Blood glucose levels were then measured at 0, 15, 30, 60, 90, and 120 minutes post-injection.

### Statistical analysis

The data presented in this study were expressed as mean values ± standard deviation (SD), with at least three independent experiments conducted for each data point. Statistical comparisons between two groups were performed using an unpaired Student's t-test. For comparisons involving more than two groups, a one-way analysis of variance (ANOVA) was employed. Differences between the experimental and control groups were considered statistically significant at *P* < 0.05.

## Results

### Catalpol mitigated triptolide-induced liver injury in mice by modulating oxidative stress and lipid peroxidation

**Figure 1:**
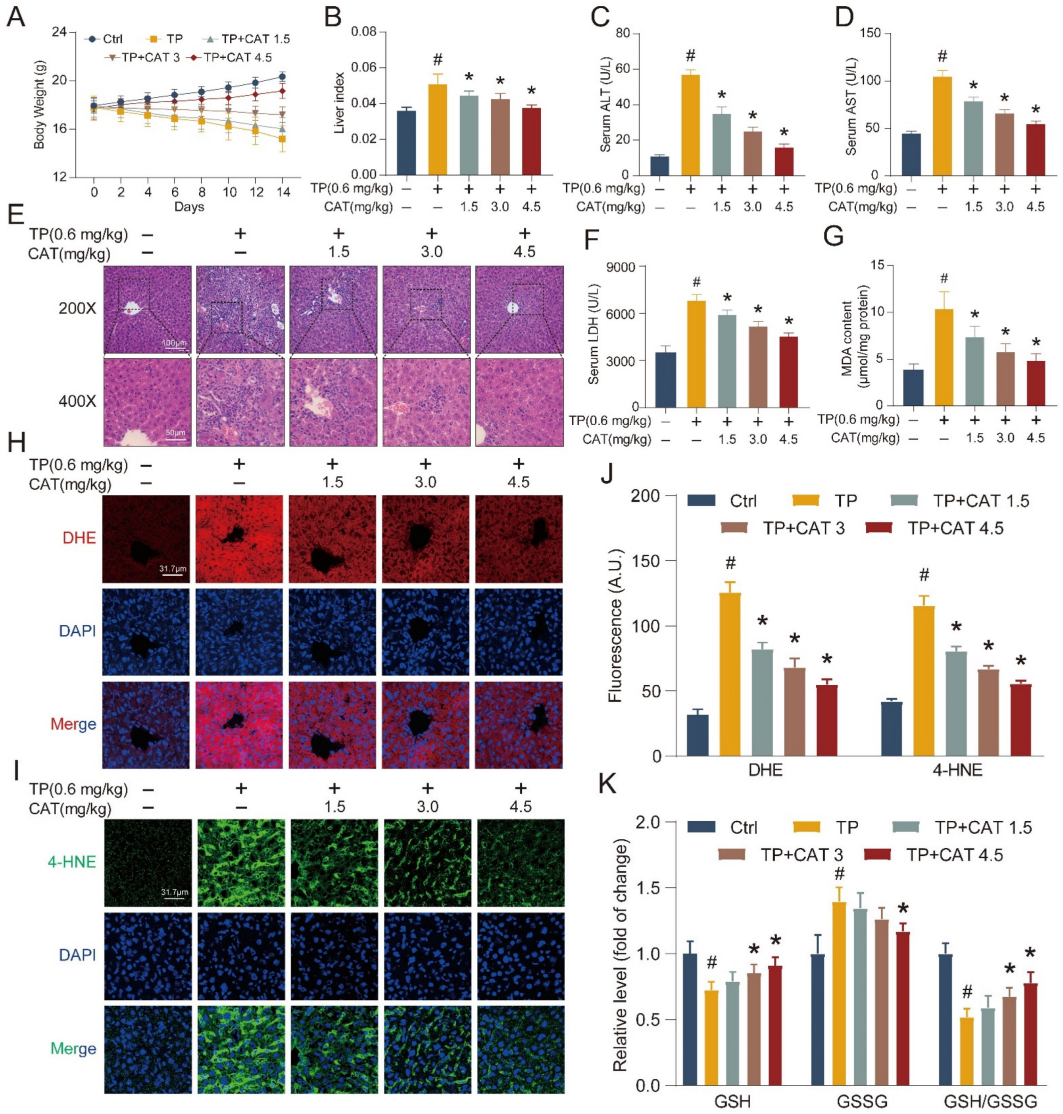
** Catalpol (CAT) mitigated triptolide (TP) induced liver injury in mice by modulating oxidative stress and lipid peroxidation.** Mice were randomly divided into the following 5 groups (six mice per group): Vehicle control (Ctrl), TP 0.6 mg/kg (TP), TP 0.6 mg/kg + CAT 1.5 mg/kg (TP + CAT1.5), TP 0.6 mg/kg + CAT 3.0 mg/kg (TP + CAT3) and TP 0.6 mg/kg + CAT 4.5 mg/kg (TP + CAT4.5). (A) Body weight. (B) Liver index. (C) Serum ALT levels. (D) Serum AST levels. (E) Microphotograph of H&E-stained sections of liver tissues (Scale bars, 100/50 μm). (F) Serum LDH levels. (G) MDA content. (H) Representative DHE fluorescence staining of liver sections for ROS production. Scale bar, 31.7 μm. (I) Immunofluorescence staining of liver sections using antibody against 4-HNE. Scale bar, 31.7 μm. (J) Quantification of DHE and 4-HNE fluorescence image. (K) The relative concentrations of GSH, GSSG and GSH/GSSG ratio measured in liver tissues. Data are expressed as mean ± SD (*n =* 6); ^#^*P* < 0.05 versus Ctrl, *P < 0.05 versus TP.

To investigate the protective effects of CAT against TP-induced liver injury, we established a model based on previous research 22-23. This model allowed us to determine the efficacy of CAT co-administration in mitigating liver damage. During the 2-week administration period, the body weight of the mice was measured every 2 days. CAT co-administration significantly reduced the weight loss induced by TP, as shown in Figure 1A. Liver weight/body weight ratio (liver index) was significantly elevated in mice subjected to TP treatment compared to the control group (Figure 1B). Different dosages of CAT combined with TP showed dose-dependent effects on body weight and liver index, with the most notable changes observed in the group receiving the highest CAT dosage (4.5 mg/kg). Serum concentrations of ALT, AST, and LDH, all markers of liver injury, were significantly increased in TP-treated mice compared to controls (Figure 1C-D, F). Importantly, CAT co-administration significantly attenuated these increases in a dose-dependent manner. Histopathological examination with H&E staining revealed severe disruption to the normal liver architecture and infiltration of inflammatory cells in the liver of mice following TP treatment for two weeks (Figure 1E). Lipid accumulation within hepatocytes was also observed. However, CAT co-administration effectively reversed these pathological changes, with the group receiving 4.5 mg/kg CAT showing the most notable improvements.

Building on the established role of oxidative stress in TP-induced liver damage 7-8, we evaluated ROS levels in our model system to investigate their contribution. DHE staining intensity, reflecting the formation of 2-hydroxyethidium, was significantly increased in the TP group compared to controls (Figure 1H, J), indicating amplified generation and dispersal of ROS within liver tissues following TP treatment. CAT co-administration reversed these effects, suggesting its ability to mitigate oxidative stress. The GSH/GSSG ratio serves as an indicator of cellular redox state. Compared to the control group, the TP group displayed a significantly lower GSH/GSSG ratio, demonstrating elevated oxidative stress and diminished antioxidant capacity in the liver (Figure 1K). Importantly, CAT treatment significantly improved the GSH/GSSG ratio in a dose-dependent manner, suggesting its effectiveness in restoring cellular redox balance. Excessive radical species can damage cellular membranes, including those of organelles, due to high levels of polyunsaturated fatty acids, leading to a process called “lipid peroxidation” 24. Lipid peroxidation can also trigger programmed cell death. We measured the hepatic levels of malondialdehyde (MDA) and 4-hydroxynonenal (4-HNE), byproducts of lipid peroxidation reactions. The findings revealed a notable rise in MDA (Figure 1G) and 4-hydroxynonenal (Figure 1I, J) production in the TP group, which was significantly diminished with CAT treatment.

### Catalpol suppressed triptolide-induced damage in hepatocytes

**Figure 2:**
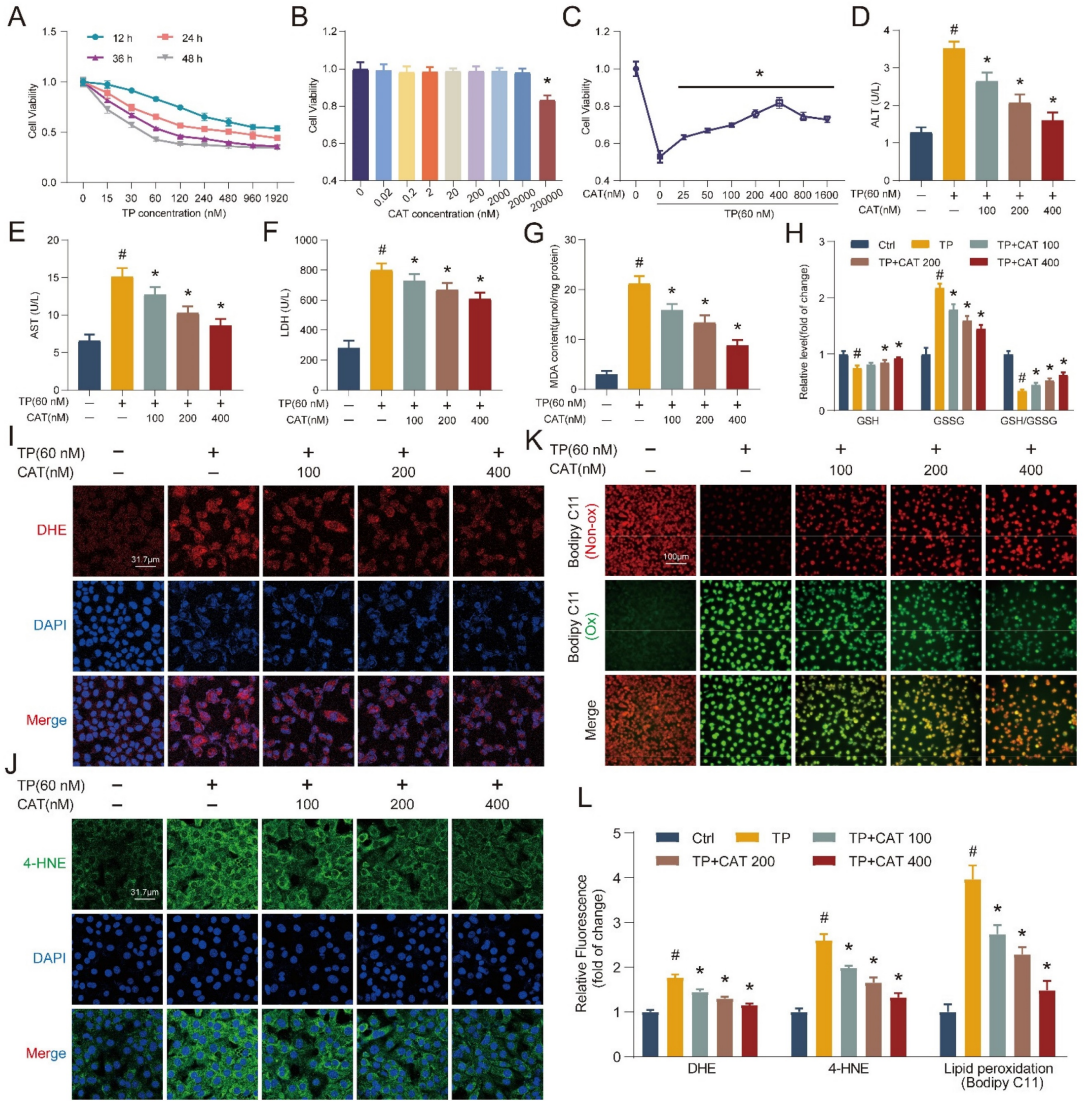
** Catalpol (CAT) suppressed triptolide (TP) induced damage in hepatocytes.** (A-C) CCK8 assay. (D-F) AML12 cells culture supernatant ALT, AST and LDH levels. (G) MDA content. (H) Relative concentrations of GSH, GSSG and GSH/GSSG ratio measured in AML12 cells. (I) Representative DHE fluorescence staining of AML12 cells. Scale bar, 31.7 μm. (J) Immunofluorescence staining of AML12 cells using antibody against 4-HNE. Scale bar, 31.7 μm. (K) Bodipy C11 staining of AML12 cells. Scale bar, 100 μm. (L) Relative quantification of DHE, 4-HNE and Bodipy C11 fluorescence images. Data are expressed as mean ± SD (*n =* 4); ^#^*P* < 0.05 versus Ctrl, *P < 0.05 versus TP.

To elucidate the protective mechanisms of CAT against TP-induced liver injury, we employed an *in vitro* model using the AML12 cell line. AML12 cells were first exposed to different dosages of TP for 12-48 h, and cell viability was assessed using the CCK8 assay (Figure 2A). Based on the CCK8 results, cells exposed to 60 nM TP for 36 h were chosen for subsequent experiments due to their appropriate stress level. A dose-response experiment with CAT demonstrated no cytotoxicity at concentrations below 20 μM (Figure 2B). Finally, cells were pre-incubated with CAT for 6 h followed by TP treatment for 36 h. The CCK8 assay revealed a concentration-dependent protective effect of CAT across the range of 100-400 nM, with optimal effects at 400 nM (Figure 2C). Therefore, subsequent experiments utilized CAT at 100, 200, and 400 nM concentrations. We measured the activities of ALT, AST, and LDH in the supernatant of AML12 cells (Figure 2D-F). CAT treatment significantly reduced the TP-induced increase in ALT, AST, and LDH in a dose-dependent manner, indicating its ability to protect cells from TP-induced cytotoxicity. Consistent with the *in vivo* findings, DHE staining and quantification of AML12 cells treated with TP and CAT (Figure 2I, L) revealed that CAT could suppress the generation of ROS triggered by TP in cellular systems. Furthermore, measurements of MDA levels (Figure 2G), the GSH/GSSG ratio (Figure 2H), and 4-HNE staining (Figure 2J, L) demonstrated that CAT reduced the TP-induced increase in lipid peroxidation levels in a concentration-dependent manner. Bodipy C11, a fluorescent ratio probe, was used to evaluate lipid peroxidation and antioxidant efficacy. Following treatment, oxidized Bodipy C11 (green) indicates lipid ROS, while non-oxidized Bodipy C11 appears red 25. As shown in Figure 2K and 2L, cells in the TP group exhibited higher levels of Bodipy C11 oxidation, while CAT treatment inhibited this effect. These results provided compelling evidence that CAT could alleviate TP-induced hepatotoxicity by improving oxidative stress and reducing lipid peroxidation.

### Catalpol improved triptolide-induced energy metabolism disorder in the liver

**Figure 3:**
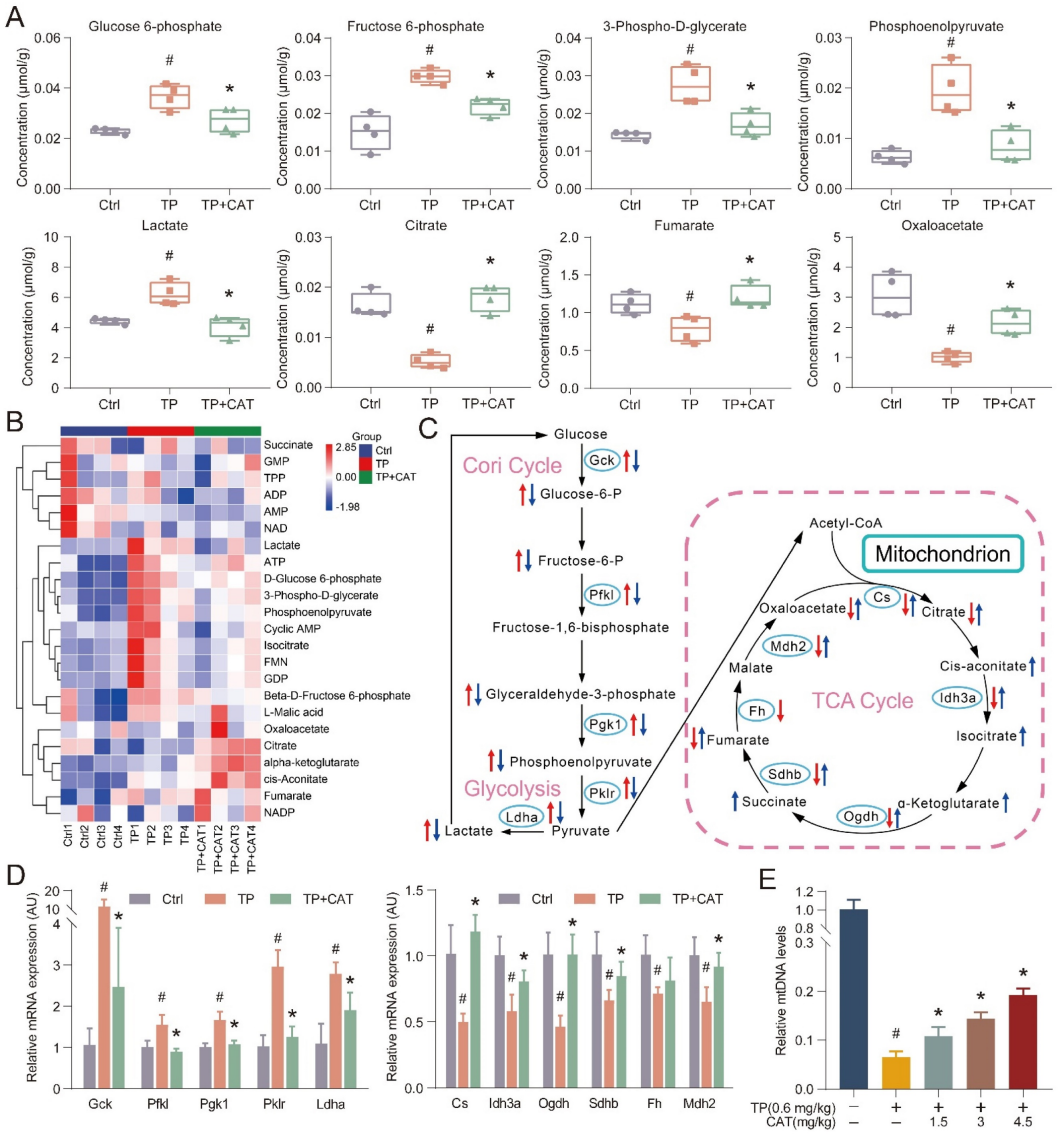
** Catalpol (CAT) improved triptolide (TP) induced energy metabolism disorder in the liver.** (A) Box plots of some significantly altered hepatic metabolites in three groups (Ctrl, TP and TP + CAT groups). (B) The hierarchical cluster analysis (HCA) presents a differentiation in the abundance of energy metabolites across the various groups. Within the heatmap, a more intense red denotes a higher level, while a deeper blue suggests a lower concentration. (C) The influence of CAT on TP-induced energy metabolic dysfunctions in the liver. Comparisons to the Ctrl group show that alterations in metabolites in the TP group are marked with a red arrow. Inversely, changes in the TP + CAT group relative to the TP group are represented with blue. An ascending arrow denotes a substantial escalation (*P* < 0.05), whilst a descending arrow indicates a noteworthy reduction (*P* < 0.05). (D) Comparative mRNA expression levels tied to energy metabolism in the livers of the three separate groups. (E) Relative mtDNA levels. Data are expressed as mean ± SD (*n =* 4); ^#^*P* < 0.05 versus Ctrl, *P < 0.05 versus TP.

The established link between energy metabolism and ROS production and the influence of redox reactions on key metabolic enzymes is well recognized. Recent research has highlighted the prominent role of energy metabolism abnormalities in the onset of TP-induced liver damage 26. Building on these findings, we employed targeted metabolomics analysis in an *in vivo* model system to detect alterations in energy metabolism that could offer valuable insights into CAT's mode of action. For streamlined data analysis, we focused on the TP + CAT group, where mice were administered with 4.5 mg/kg of CAT and 0.6 mg/kg of TP. PCA was employed to visually represent and distinguish sample grouping, patterns, and outliers in the data set. The PCA score plots (Figure S1A) revealed distinct metabolic profiles among the Ctrl, TP, and TP + CAT groups. Notably, samples from the Ctrl and TP groups showed clear differentiation, suggesting that TP exposure disrupted the normal metabolic profile of mice. Conversely, the TP + CAT group displayed a closer resemblance to the control group, indicating favorable quality parameters. Hierarchical cluster analysis (HCA) was used to visualize the abundance of each metabolite through a heatmap, where each cell's color signified the respective metabolite's concentration value (Figure 3B). Although variations in color intensity were observed across samples from different groups, the four biological duplicates from each group demonstrated consistent clustering patterns, highlighting the reproducibility of the data. Overall, the findings from PCA and HCA demonstrated high consistency. Upon analysis of metabolite changes in various groups (Figure 3A, S1B-C), we observed that the TP group exhibited notable alterations in 13 energy metabolites within the liver compared to the Ctrl group. These metabolites included such as glucose 6-phosphate, fructose 6-phosphate, lactate, and citrate. Importantly, these alterations were significantly mitigated following CAT treatment.

Metabolomic analysis indicated that CAT modulated glycolysis and the TCA cycle in response to TP-induced liver energy disruption, likely through the regulation of key enzymes in these metabolic pathways. To investigate this hypothesis, we measured the mRNA expression levels of key regulatory enzymes using RT-qPCR to assess the protective effects of CAT on TP-induced liver energy disorders (Figure 3D). Compared to the control group, the TP group displayed significantly increased expression of crucial glycolytic enzymes such as Gck, Pfkl, Pgk1, Pklr, and Ldha. Conversely, these enzyme levels significantly decreased following CAT treatment. Similarly, the mRNA expression levels of Cs, Idh3a, Ogdh, Sdhb, Fh, and Mdh2 in the TCA cycle were significantly downregulated in the TP group compared to the control group but underwent significant upregulation after CAT intervention. Figure 3C illustrates the alterations in energy metabolites and metabolic enzymes, suggesting that TP induced an increased glycolytic flux in the murine liver, accompanied by a reduction in aerobic oxidation. Importantly, CAT treatment effectively restored normal metabolic homeostasis. Mounting evidence highlights the importance of mitochondria in cellular metabolism, with their dysfunction potentially leading to metabolic disorders. The abundance of mtDNA serves as an indicator of mitochondria within each cell and can be harnessed to quantify damage 27. As illustrated in Figure 3E, the TP group exhibited a significant decrease in mtDNA copy number, a marker of mitochondrial biogenesis. Conversely, CAT treatment resulted in a dose-dependent increase in mtDNA copy number. In summary, our findings demonstrate that CAT exerted significant hepatoprotective effects by ameliorating TP-induced energy metabolism disorders and mitochondrial dysfunction.

### The beneficial role of catalpol was related to regulating the balance between glycolysis and OXPHOS

**Figure 4:**
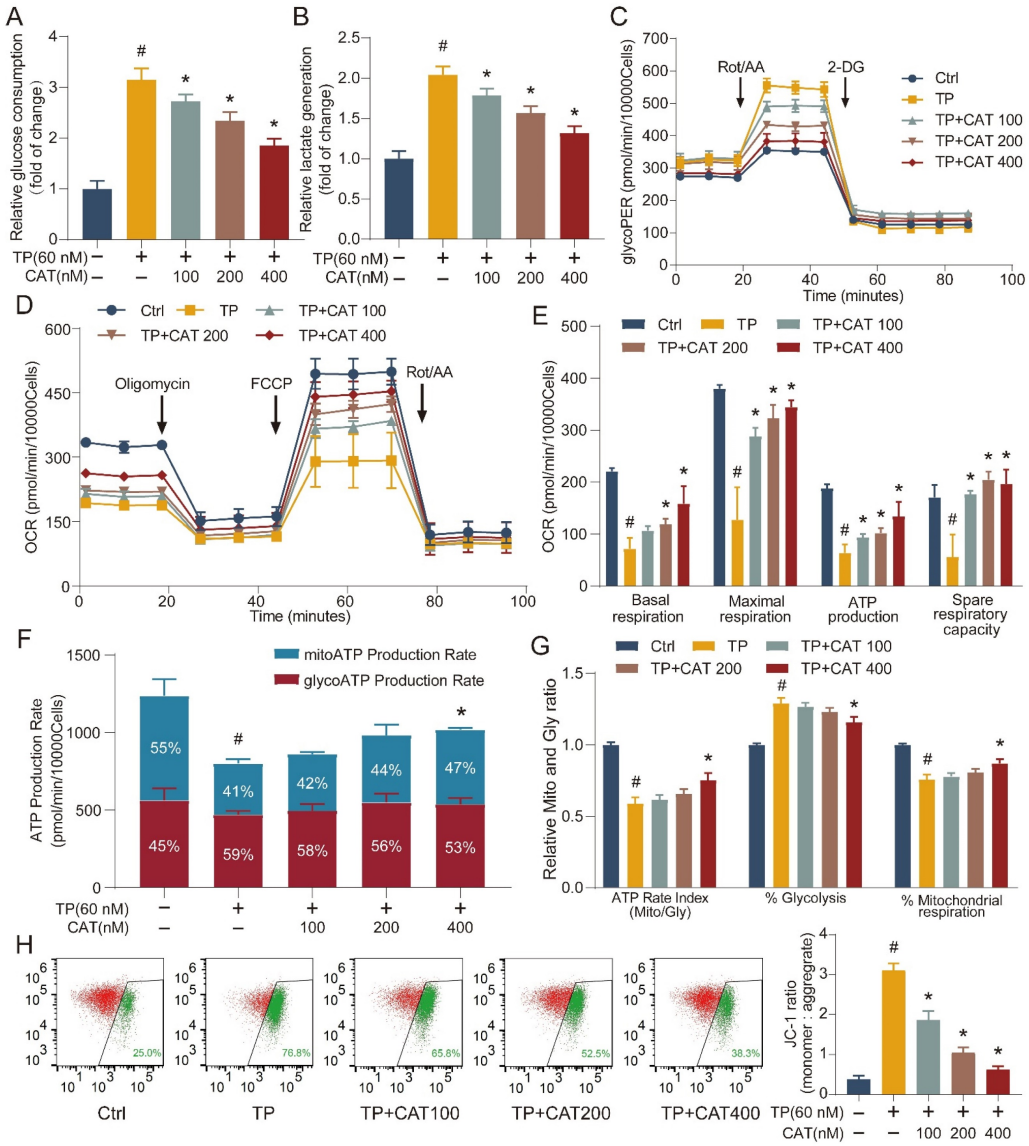
** The beneficial role of catalpol (CAT) was related to regulating the balance between glycolysis and OXPHOS.** (A) Relative glucose consumption. (B) Relative lactate generation. (C) Glycolytic rate assay measuring glycolytic proton efflux rate (glycoPER). (D) Assessment of mitochondrial stress through direct quantification of the oxygen consumption rate (OCR). (E) Outcomes from the quantitative analysis of cellular respiration parameters: basal respiration, maximal respiration, ATP production, and spare respiratory capacity. (F) Real-time ATP production rate test calculated ATP manufacture rate from glycolysis (Gly) and mitochondrial respiration (Mito). (G) Mito/Gly ATP rate index, % Glycolysis and % Mitochondrial respiration were conducted. (H) Flow cytometry inspection of the JC-1 experiment (mitochondrial membrane potential) in AML12 cells. Rot/AA: Rotenone plus Antimycin A; 2-DG: 2-deoxy-D-glucose. Data are expressed as mean ± SD (*n =* 3); ^#^*P* < 0.05 versus Ctrl, *P < 0.05 versus TP.

Building on the established role of mitochondria in energy metabolism, we employed the Seahorse Extracellular Flux Analyzer (XFe24) to elucidate the influence of CAT on TP-mediated disruption in cellular energy metabolism. This instrument enables real-time monitoring of energy metabolism in live cells. We first evaluated the impact of TP and TP + CAT administration on cellular glucose uptake and lactate production. The results shown in Figure 4A-B suggest that the TP group exhibited increased glucose uptake and lactate production levels compared to the control group. These findings indicate enhanced cellular glycolysis in the TP group. After measuring the glycolytic rate, we observed that the TP group displayed a significantly higher glycolytic proton efflux rate (Figure 4C), basal glycolysis, basal proton efflux rate, and compensatory glycolysis, while acidification after 2-deoxyglucose (2-DG) treatment remained stable (Figure S2A-D). These results suggest that TP enhanced cellular glycolytic capacity, an effect that could be reversed by CAT in a concentration-dependent manner. The oxygen consumption rate serves as a direct indicator of mitochondrial electron transfer rate, providing a real-time measure of cellular mitochondrial function. In our study, the TP group exhibited a noticeable reduction in basal respiration, maximal respiration (influenced by FCCP), ATP production, and spare respiratory capacity, which reflects the ability of mitochondria to respond to increased energy demands, compared to the control group. However, we observed no significant changes in non-mitochondrial respiration or coupling efficiency (Figure 4D-E, S2E-F). CAT treatment significantly improved TP-induced mitochondrial respiratory impairment. Additionally, compared to the control group, the TP group displayed a diminished rate of ATP production from mitochondrial respiration (Mito) and a reduced overall ATP production rate, at 49.1 ± 4.2% and 64.7 ± 3.7% of the control group, respectively. Furthermore, the ratio of aerobic respiration to glycolytic ATP production significantly declined to 58.9 ± 4.4%. The contribution of glycolysis to ATP production increased notably by 28.9 ± 4%, whereas the contribution of aerobic respiration drastically reduced by 24.1% ± 3.4%. However, after CAT treatment, the form of ATP production induced by TP returned to normal (Figure 4F-G). Building on the observed mitochondrial dysfunction, we investigated the influence of TP and CAT on ΔΨm. Established research demonstrates that ΔΨm, generated by proton pumps (Complexes I, III, and IV) within the respiratory chain, is crucial for the energy storage mechanism of OXPHOS. Along with the proton gradient, ΔΨm drives the movement of protons across the membrane, creating a proton gradient which fuels ATP synthesis. Consequently, ΔΨm is indispensable for ATP generation, and decreased ΔΨm disrupts ATP production. JC-1, a fluorescent cationic dye, was used to evaluate ΔΨm by measuring the red-to-green fluorescence intensity ratio from cells stained with JC-1. As depicted in Figure 4H, TP treatment caused a decrease in ΔΨm. Conversely, CAT treatment increased ΔΨm in a concentration-dependent manner. These findings suggest that TP treatment triggered a shift in primary energy metabolism from the respiratory chain to glycolysis for ATP generation. CAT application, by restoring ΔΨm, could potentially restore the balance between glycolysis and OXPHOS.

### Catalpol alleviated triptolide-induced glycogen metabolism and gluconeogenesis disorders through the SIRT1/HIF-1α pathway

**Figure 5:**
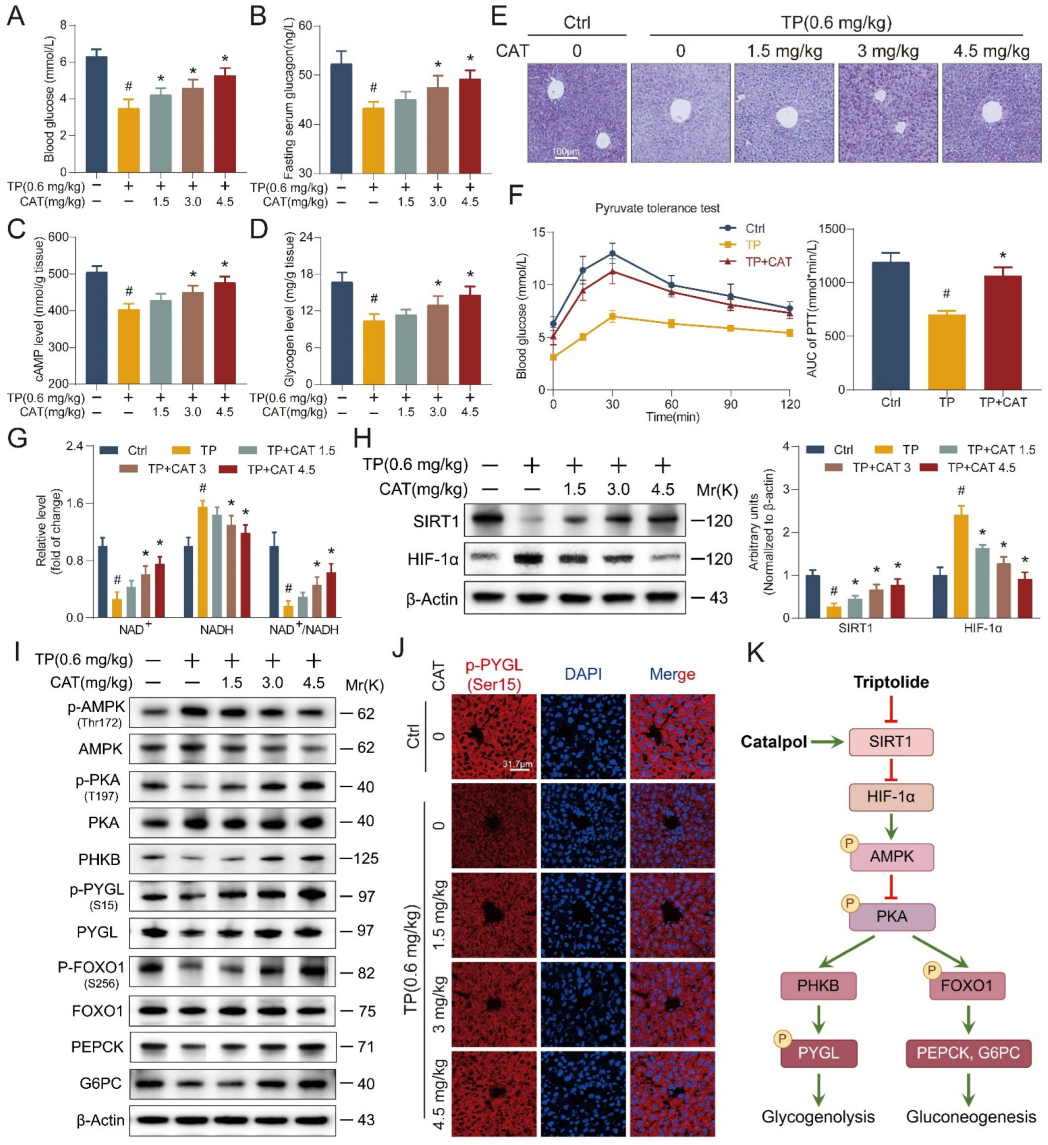
** Catalpol (CAT) alleviated triptolide (TP) induced glycogen metabolism and gluconeogenesis disorders through the SIRT1/HIF-1α pathway.** (A) Blood glucose. (B) Fasting serum glucagon levels. (C) Hepatic cAMP levels. (D) Hepatic glycogen levels. (E) Microphotograph of PAS-stained sections of liver tissues. Scale bar, 31.7 μm. (F) Following a 16-hour fasting period, the mice were administered an intraperitoneal injection of sodium pyruvate at a dosage of 1.5 g/kg body weight. Subsequently, their blood glucose levels were recorded at predetermined time intervals. The values for the area under the curve (AUC) of blood glucose were then computed. (G) The relative concentrations of NAD^+^, NADH and NAD^+^/NADH ratio measured in liver tissues. (H) Western blot analysis of SIRT1 and HIF-1α expression from mouse liver. (I) The expression levels of glycogenolysis and gluconeogenesis related proteins. (J) Immunofluorescence staining of liver sections using antibody against p-PYGL (Ser15). Scale bar, 31.7 μm. (K) Schematic of signaling pathway changes. Data are expressed as mean ± SD (*n =* 6); ^#^*P* < 0.05 versus Ctrl, *P < 0.05 versus TP.

The liver plays a central role in maintaining glucose homeostasis. Hypoglycemia is a common complication of DILI, characterized by disrupted gluconeogenesis and increased extrahepatic glucose utilization 28. This effect has also been observed in TP-induced liver injury 23. Our findings suggest that CAT might have a protective effect against TP-induced hypoglycemia (Figure 5A). Tight control of blood glucose levels necessitates the antagonistic and synergistic effects of glucagon and insulin. We measured fasting serum insulin and glucagon levels in five distinct mouse cohorts (Figure 5B, S3A). Compared to the control group, the TP group exhibited a modest decrease in serum insulin levels and a significant reduction in glucagon levels. Conversely, CAT treatment resulted in a dose-dependent increase in glucagon levels. Glucagon binds to liver receptors, stimulating the production of cAMP, which activates liver phosphorylase to convert glycogen to glucose. It can also stimulate hepatic gluconeogenesis by activating protein kinase A (PKA) through cAMP 29. We measured the cAMP levels in the mice's livers and observed that CAT administration notably reversed the TP-induced reduction of cAMP levels (Figure 5C). During fasting, the liver primarily synthesizes glucose through gluconeogenesis and breaks down glycogen to regulate blood sugar levels. PAS staining demonstrated a significant decrease in liver glycogen levels in the TP group compared to the control group. However, this decline was reversed with CAT treatment (Figure 5D-E). To elucidate the mechanism by which CAT reversed the TP-induced decrease in mouse blood glucose levels, we conducted an *in vivo* pyruvate tolerance test to measure hepatic gluconeogenesis. Figure 5F illustrated that post-pyruvate injection, blood glucose levels showed a significant rise in the control group of mice. However, this rise was not observed in the TP group, indicating impaired hepatic gluconeogenesis following TP treatment. Conversely, CAT demonstrated a significant restorative effect. Furthermore, the IPGTT was employed to assess peripheral glucose uptake. The findings revealed no substantial alterations in blood glucose levels in all three groups post-glucose challenge under fasting conditions (Figure S3B), suggesting that peripheral glucose uptake was not impeded by either TP or CAT treatment.

Building on the observed effects on glucose homeostasis, we investigated the expression of SIRT1 and HIF-1α, two proteins known to be involved in TP-induced liver damage 30-31. SIRT1, a highly conserved NAD^+^-dependent histone deacetylase, has been implicated in various stress responses, while HIF-1α plays a critical role in regulating oxygen homeostasis under conditions like hypoxia, inflammation, and oxidative stress. It also regulates cellular metabolism.

First, we determined the hepatic NAD^+^/NADH ratio (Figure 5G). The results showed a significant decrease in NAD^+^ levels in the TP group compared to the control group, with a concomitant increase in NADH content. The administration of CAT effectively reversed this alteration. Furthermore, the study of SIRT1/HIF-1α gene and protein expression (Figure 5H and S3C-D) indicated a decrease in SIRT1 and an increase in HIF-1α expression in the liver of the TP group mice. Concurrently, the application of CAT significantly counteracted this effect in a dose-dependent manner.

To elucidate the mechanisms underlying the observed effects on glucose metabolism, Western blot analysis was performed to identify key proteins associated with hepatic glycogen breakdown and gluconeogenesis signaling pathways. Previous research has shown that TP disrupts the balance between glycolysis and OXPHOS, leading to diminished intracellular ATP synthesis. This, in turn, results in elevated levels of adenosine monophosphate (AMP) and adenosine diphosphate (ADP), which activate AMP-activated protein kinase (AMPK) 32. Activated AMPK, along with AMP, subsequently downregulates cAMP production by activating phosphodiesterase 4B (PDE4B) and inhibiting adenylate cyclase, thereby attenuating PKA signaling 33. In the liver, PKA exerts control over critical processes in glucose metabolism through the phosphorylation of various downstream targets. Notably, PKA can activate phosphorylase kinase (PHKB) and phosphorylate glycogen phosphorylase (PYGL) at the Ser15 site, thereby initiating glycogen degradation 34. Additionally, PKA directly phosphorylates FoxO1, leading to increased transcription of phosphoenolpyruvate carboxykinase (*PEPCK*) and glucose-6-phosphatase (*G6PC*), enzymes that promote gluconeogenesis 35. Western blot analysis (Figure 5I and S3E-F) revealed a significant reduction in PKA phosphorylation in the liver of TP-treated mice, accompanied by decreased levels of downstream proteins PHKB and phosphorylated PYGL (Ser15). These findings suggest impaired glycogen degradation. Conversely, CAT administration resulted in a dose-dependent enhancement of glycogen degradation. These results are further corroborated by the immunofluorescence data presented in Figure 5J. Furthermore, TP treatment led to decreased levels of p-FoxO1 (S256) in the liver, thereby restraining gluconeogenesis through the downregulation of PEPCK and G6PC expression (Figure 5K). To gain a more comprehensive understanding of the effects of TP on hepatic glycogen metabolism, we examined the mRNA expression of key genes involved in this process (Figure S3G-H) 36. These genes encode enzymes critical for both glycogen synthesis (Gys2, Pgm1, Pgm2, Pgm3, Ugp, Gyg2, Gbe1) and breakdown (Pygl, Phka2, Phkb, Phkg2, Pck1, G6pt). Our findings suggest that TP treatment suppressed hepatic glycogen metabolism, as evidenced by the altered expression of these genes. In conclusion, CAT treatment effectively ameliorated TP-induced glucose metabolism disruption in mice. This protective effect likely stems from CAT's ability to enhance glycogen metabolism and gluconeogenesis in the liver.

### SIRT1/HIF-1α as a target of catalpol: influence of *SIRT1* overexpression or knockout on its beneficial effects *in vitro*

**Figure 6:**
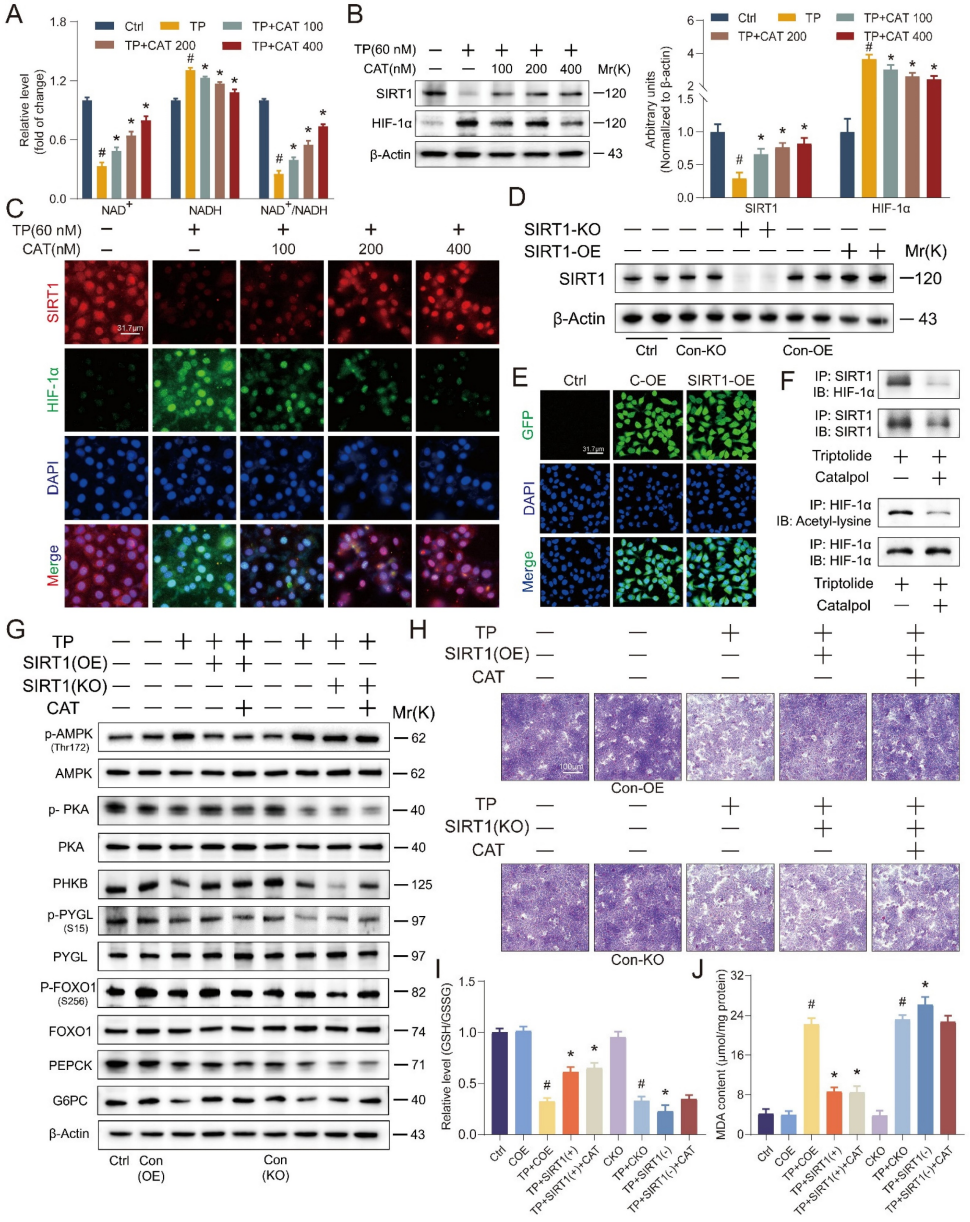
** SIRT1/HIF-1α was the target of catalpol (CAT) and the beneficial effects of CAT were influenced by the overexpression or knockout of SIRT1 *in vitro*.** (A) The relative concentrations of NAD^+^, NADH and NAD^+^/NADH ratio measured in AML12 cells. (B) Western blot analysis of SIRT1 and HIF-1α expression from AML12 cells. (C) Immunofluorescence staining of AML12 cells using antibodies against SIRT1 and HIF-1α. Scale bar, 31.7 μm. (D) SIRT1 protein expression of transfected cells. (E) Transfection results of AML12 cells. Scale bar, 31.7 μm. (F) Co-immunoprecipitation was done using equal protein quantities with either SIRT1 antibody or HIF-1α antibody, followed by immunoblotting procedure using antibodies against SIRT1, HIF-1α or Acetyl-lysine, illustrating the impact of TP and CAT. (G) The levels of proteins associated with glycogenolysis and gluconeogenesis in the transfected cells. (H) Microphotograph of transfected cells stained with PAS. Scale bar, 100 μm. (I) Relative determination of GSH/GSSG ratio in transfected cells. (J) MDA content of transfected cells. Data are expressed as mean ± SD (*n =* 3); ^#^*P* < 0.05 versus Ctrl, COE or CKO, *P < 0.05 versus TP, TP + COE or TP + CKO.

*In vitro* studies using AML12 cells were conducted to evaluate the effects of TP and CAT on the SIRT1/HIF-1α signaling pathway, and the findings mirrored our *in vivo* observations (Figure 6A-B). We observed similar changes in the ratios of NAD^+^/NADH and SIRT1/HIF-1α protein expression. Immunofluorescence results (Figure 6C) corroborated these findings, revealing consistent changes and co-localization of SIRT1 and HIF-1α within the cell nucleus. These observations suggest a potential role for SIRT1/HIF-1α in the protective effects of CAT against TP-induced liver damage. To elucidate the role of SIRT1 in CAT's protective effects, AML12 cells were transfected with lentiviruses carrying *SIRT1* overexpression (SIRT1-OE) or knockout (SIRT1-KO) constructs, along with a control vector. The transfection efficiency was confirmed by green fluorescence imaging and Western blotting (Figure 6D-E, S4B). Previous studies suggest that SIRT1 regulates HIF-1α by facilitating the formation of protein complexes and directly promoting its deacetylation, leading to its deactivation 37. Co-immunoprecipitation was employed to assess the interaction between SIRT1 and HIF-1α, while an anti-acetyl-lysine antibody was used to evaluate HIF-1α acetylation status (Figure 6F and S4A). TP treatment increased endogenous HIF-1α production and the binding between SIRT1 and HIF-1α was observed. Conversely, co-treatment with CAT and SIRT1-OE lentivirus reduced this interaction. *SIRT1* knockout increased HIF-1α acetylation, whereas CAT and *SIRT1* overexpression significantly reversed this effect. These data suggest that TP-induced downregulation of SIRT1 promotes HIF-1α acetylation, leading to disruptions in energy metabolism. Conversely, CAT treatment can yield a significant counteracting effect.

To further investigate the functional relationship between SIRT1 and CAT, we assessed their combined effects on glucose metabolism and oxidative stress in AML12 cells. *SIRT1* overexpression (SIRT1-OE) significantly increased cellular glycogen content compared to *SIRT1* knockout (SIRT1-KO) cells (Figure 6H, S4C). Importantly, the beneficial effect of CAT on glycogen levels was diminished in the absence of SIRT1. Similarly, analysis of glycogen breakdown and gluconeogenesis (Figure 6G, S4D-J) revealed that *SIRT1* overexpression effectively mitigated TP-induced alterations in glucose metabolism. Notably, the stimulatory effect of CAT on proteins involved in this pathway was was negligible in SIRT1-KO cells but substantial in SIRT1-OE cells.

These findings suggest that SIRT1 is likely required for CAT's protective effects on glucose metabolism. We further hypothesized that CAT's protective action against TP-induced hepatocyte injury might be inhibited in SIRT1-KO cells. Thus, when this compound displayed its advantageous impacts, SIRT1 could possibly be a target of CAT. Next, we examined the impact of SIRT1 on CAT-mediated reduction of oxidative stress. Levels of ROS accumulation and lipid peroxidation, as measured by GSH/GSSG ratio, MDA content, DHE staining, and 4-HNE staining (Figure 6I-J, S5A-D), were significantly higher in SIRT1-KO cells compared to SIRT1-OE cells. Importantly, the effectiveness of CAT in mitigating these markers of oxidative stress was diminished in the absence of SIRT1, while SIRT1 overexpression further enhanced CAT's protective effects. In conclusion, these data demonstrate a functional interplay between SIRT1 and CAT. SIRT1 is essential for CAT's protective effects on glucose metabolism and oxidative stress in AML12 cells, suggesting a potential mechanism by which CAT exerts its hepatoprotective function.

### Liver-specific *SIRT1* knockout aggravated triptolide-induced liver injury and weakened the beneficial effects of catalpol

**Figure 7:**
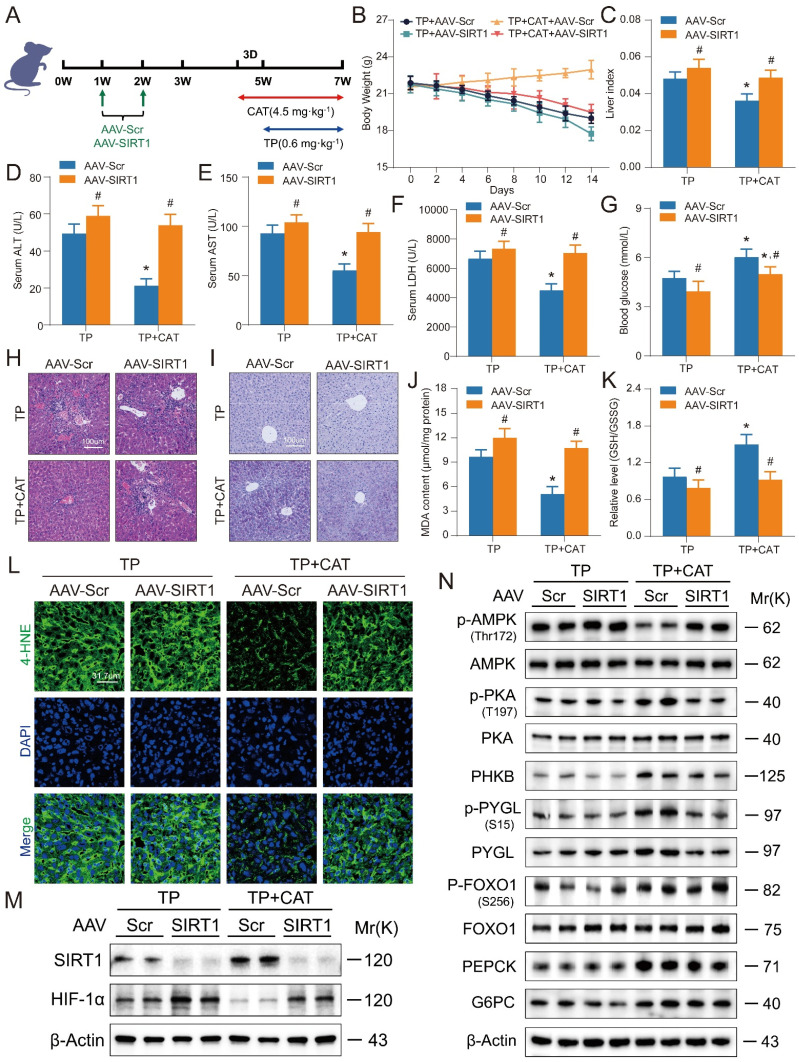
** Liver-specific *SIRT1* knockout aggravated triptolide (TP) induced liver injury and weakened the beneficial effects of catalpol (CAT).** (A) Experimental flow chart. (B) Body weight. (C) Liver index. (D-F) Serum ALT, AST and LDH levels. (G) Blood glucose levels. (H) Microphotograph of H&E-stained sections of liver tissues. Scale bar, 31.7 μm. (I) Microphotograph of PAS-stained sections of liver tissues. Scale bar, 31.7 μm. (J) MDA content. (K) Relative determination of GSH/GSSG ratio in liver tissues. (L) Immunofluorescence staining of liver sections using antibody against 4-HNE. Scale bar, 31.7 μm. (M) The protein expression levels of SIRT1 and HIF-1α. (N) The expression levels of glycogenolysis and gluconeogenesis related proteins. Data are expressed as mean ± SD (*n =* 6); An “*” indicates significant difference between TP and TP + CAT groups (*P* < 0.05). A “#” indicates significant difference between AAV-Scr and AAV-SIRT1 in either TP or TP + CAT groups (*P* < 0.05).

To validate the role of the SIRT1/HIF-1α pathway in mediating CAT's protective effects against TP-induced liver damage, we employed SIRT1 CRISPR All-in-One AAV Vector packaged virus for liver-specific *SIRT1* knockout in mice via tail vein injection (Figure 7A). Immunofluorescence and western blot analysis confirmed successful *SIRT1* deletion (Figure S6A-B). Subsequently, mice were treated with TP alone or in combination with CAT. Body weight was monitored every two days. *SIRT1* knockout significantly exacerbated TP-induced weight loss, and this effect was not reversed by CAT treatment (Figure 7B). Liver index, a marker of liver enlargement, was significantly higher in the TP + CAT + AAV-SIRT1 group compared to the TP + CAT + AAV-Scr group (Figure 7C). This finding suggests that *SIRT1* deficiency abrogated the protective effect of CAT on liver weight. Similarly, serum ALT, AST, and LDH activities, all indicators of liver damage, were significantly elevated by *SIRT1* knockout, particularly in the CAT-treated group (Figure 7D-F). Histopathological analysis using H&E staining corroborated these findings (Figure 7H). *SIRT1* knockout intensified inflammatory cell infiltration and enlargement, further highlighting the diminished protective effect of CAT in the absence of SIRT1. Consistent with these observations, PAS staining and glycogen content analysis (Figure 7I, S6C) revealed that CAT treatment failed to improve the significant decrease in liver glycogen content in the TP + CAT + AAV-SIRT1 group. Furthermore, biochemical analysis demonstrated that while CAT treatment reversed the TP-induced decrease in blood glucose levels, this protective effect was significantly attenuated by *SIRT1* knockout (Figure 7G). Evaluation of oxidative stress markers revealed that *SIRT1* knockout abolished the protective effects of CAT. Compared to the TP + AAV-Scr group, the TP + CAT + AAV-Scr group exhibited a significant increase in the liver's GSH/GSSG ratio, indicating enhanced antioxidant capacity, along with a substantial decrease in ROS levels (Figure 7K, S6D, F). Conversely, no significant changes were observed in the TP + CAT + AAV-SIRT1 group. Lipid peroxidation markers, MDA and 4-HNE, displayed a similar trend (Figure 7J, L). These findings suggest that SIRT1 deficiency abrogated CAT's ability to mitigate TP-induced oxidative stress. Western blot analysis of the SIRT1/HIF-1α pathway and proteins associated with glycogen breakdown and gluconeogenesis (Figure 7M-N, S6E-I) revealed that TP significantly elevated hepatic HIF-1α levels in *SIRT1* knockout mice, leading to decreased expression of proteins involved in gluconeogenesis and glycogen breakdown. Notably, CAT treatment partially mitigated the glucose metabolism dysfunction induced by *SIRT1* knockout. In conclusion, these data demonstrate that SIRT1 is essential for CAT's protective effects against TP-induced liver damage in mice. *SIRT1* deficiency significantly attenuated CAT's ability to improve liver function, glucose metabolism, and oxidative stress response.

## Discussion

The global rise in autoimmune diseases necessitates the development of affordable and effective therapeutic interventions 38. Triptolide, a potent bioactive compound isolated from the traditional Chinese medicinal plant TwHf, possesses remarkable immunosuppressive and anti-inflammatory properties. However, despite its potential for treating various autoimmune conditions, TP's clinical application is hampered by its significant toxicity profile. While several antioxidant compounds, including ginsenoside Rb1, chlorogenic acid, and isoliquiritigenin, have shown efficacy in alleviating TP-induced liver injury 39-41, further research suggests that this injury often coincides with mitochondrial dysfunction and disrupted energy metabolism, both of which are closely linked to oxidative stress-induced damage. Therefore, exploring and developing novel strategies to mitigate or prevent TP-induced hepatotoxicity is crucial.

Catalpol, a potent antioxidant derived from *Rehmannia glutinosa* (Gaertn.) DC., has been shown to improve liver glucose metabolism in diabetic conditions and enhance the activities of antioxidant enzymes superoxide dismutase, catalase, and glutathione peroxidase in the liver 42. Compelling evidence also supports the protective role of CAT in mitigating TP-induced liver damage. Previous studies suggest that CAT suppresses excessive autophagy through the PERK-ATF4-CHOP pathway and synergistically activates Phase I and Phase II detoxifying enzymes (CYP3A2/4, CYP2C9, and UGT1A6) via the CAR and NRF2 pathways, ultimately attenuating TP-induced liver toxicity 17, 18. However, the precise mechanisms by which CAT alleviates TP-induced disruptions in hepatic glucose metabolism and oxidative stress remain unclear.

DILI pathogenesis is significantly driven by oxidative stress and lipid peroxidation, which arise from an imbalance between free radicals and antioxidants 28. This imbalance damages liver cells through lipid peroxidation. Histologically, DILI manifests primarily as punctate necrosis, hepatocyte loss, infiltration of mixed inflammatory cells (predominantly eosinophils and neutrophils) surrounding the nucleus, and often an elevated liver index 43. Our H&E staining results corroborated these hallmarks, demonstrating that CAT treatment significantly mitigated TP-induced hepatocellular necrosis and inflammatory cell infiltration. Clinically, liver injury is often assessed using biomarkers like ALT, AST, and LDH. Consistent with this approach, our study observed a significant increase in these enzyme activities in the TP group, with levels decreasing upon CAT treatment. Furthermore, both in mice and AML12 cells, TP treatment induced significant oxidative stress and lipid peroxidation. However, evaluation of markers like DHE and 4-HNE staining, along with GSH/GSSG and MDA levels, confirmed CAT's effectiveness in alleviating these TP-induced effects.

DILI-induced mitochondrial injury disrupts various aspects of hepatic function, particularly glucose metabolism. Metabolomic studies have shown that excessive acetaminophen (APAP) depletes liver glucose and glycogen stores, likely due to impaired mitochondrial function and a compensatory upregulation of glycolysis 44. TP appears to exert similar effects 23. Our findings from PAS staining and blood glucose analysis revealed that CAT treatment effectively mitigated TP-induced glycogen depletion and hypoglycemia. Furthermore, CAT treatment ameliorated TP-induced glucose metabolism dysfunction by enhancing both glycogen metabolism and gluconeogenesis, as evidenced by changes in the expression of key genes involved in these processes. To elucidate CAT's role in regulating blood glucose, we examined hormone levels (insulin and glucagon) and protein expression of key enzymes in glycogen breakdown (PHKB, p-PYGL) and gluconeogenesis (p-FOXO1, PEPCK, G6PC). These analyses suggest that CAT significantly promoted glycogenolysis and gluconeogenesis. An intriguing observation was the decrease in glycogen breakdown despite glycogen depletion following TP treatment. This discrepancy may be attributed to potential abnormalities in glucuronidation during drug metabolism 45 or the influence of TP and its metabolites on glycogen synthesis through modulation of inflammatory responses or cellular signaling pathways 46. Further investigation is warranted to understand the mechanisms underlying the remarkable restorative effect of CAT on TP-induced reductions in glucagon levels.

Disruptions in energy metabolism play a critical role in the progression of TP-induced liver damage. This imbalance disrupts hepatic glucose and lipid homeostasis, increasing ROS production, lipid peroxidation, cytokine release, and cell death. Cells rely primarily on glycolysis and OXPHOS for ATP production. Studies have shown that TP inhibits OXPHOS, leading to NAD^+^ depletion and reduced pyruvate oxidation by the pyruvate dehydrogenase complex. Consequently, the decreased NAD^+^/NADH ratio promotes the conversion of pyruvate to lactate, thereby enhancing glycolysis, as evidenced by elevated serum lactate/pyruvate ratio and hyperlactatemia 9. To investigate these changes, we assessed glycolysis and OXPHOS activity in both animal and cell models. We observed increased glycolytic enzyme activity, decreased activity of mitochondrial enzymes, and reduced total ATP generation in the TP group. Furthermore, the decline in mtDNA and ΔΨm in the TP group correlated with the observed changes in ROS, OXPHOS, and ATP levels. Importantly, CAT administration effectively restored the balance between glycolysis and OXPHOS, leading to increased ATP levels, decreased ROS production, and enhanced mtDNA copy number and ΔΨm. These findings suggest that CAT treatment improves energy metabolism and promotes mitochondrial biogenesis.

DILI pathogenesis can involve specific drugs or their metabolites inducing significant oxidative stress, leading to a hypoxic cellular environment. This hypoxia triggers the upregulation of HIF-1α expression, initiating hypoxia-related responses. Furthermore, acetylation of HIF-1α can enhance its transcriptional activity, potentially intensifying cellular stress responses and exacerbating liver damage 47. SIRT1, a NAD⁺-dependent protein deacetylase, removes acetyl groups from histones within specific gene promoter regions, leading to gene silencing 48. While primarily nuclear, SIRT1 also exhibits some cytoplasmic localization. The specific subcellular distribution depends on the relative strength of nuclear localization and export signals, which can vary by cell type and environmental cues 49. Our findings indicate that CAT treatment significantly increases SIRT1 protein levels and suppresses HIF-1α expression. Further investigation revealed a direct interaction between SIRT1 and HIF-1α, with SIRT1 modulating HIF-1α activity by deacetylating specific lysine residues. Interestingly, studies have shown a link between the glycolysis-OXPHOS balance and the expression of SIRT1/HIF-1α 50. Therefore, CAT's effectiveness in alleviating TP-induced liver energy stress might be partially attributed to its modulatory effects on the SIRT1/HIF-1α pathway.

To validate our hypothesis regarding SIRT1's role, we employed two approaches: 1) lentiviral transfection in AML12 cells to achieve either *SIRT1* overexpression or knockout, and 2) SIRT1-CRISPR AAV virus administration in mice for liver-specific *SIRT1* knockout. Consistent methodologies (as described previously) were used for sample collection and analysis. As expected, SIRT1 expression levels significantly correlated with energy metabolism, glycogen depletion, oxidative stress, and lipid peroxidation. The absence of SIRT1 exacerbated TP-induced hepatocyte damage, while *SIRT1* overexpression mitigated this effect. Furthermore, contrasting the effects of CAT under varying SIRT1 expression levels revealed that *SIRT1* knockout significantly blunted CAT's ability to alleviate TP-induced liver injury. These findings suggest a critical interplay between SIRT1 and CAT in ameliorating hepatic glucose metabolism dysfunction and oxidative stress. Our data imply that CAT may function as a SIRT1 activator, potentially influencing the SIRT1/HIF-1α pathway and mitigating TP-induced liver damage progression. This represents a novel mechanism by which CAT exerts its protective effects. The precise mechanism of CAT-mediated SIRT1 activation remains under investigation. A possible explanation is that after CAT forms a stable binding with SIRT1, it leads to structural modifications in SIRT1, making it easier to bind to substrates and enhancing its deacetylase activity 51.

In summary, this study demonstrates that CAT effectively mitigates TP-induced liver injury in mice and hepatotoxicity in AML12 cells. CAT treatment also improves glucose metabolism dysfunction and oxidative stress caused by mitochondrial dysfunction. Notably, CAT administration significantly enhances mitochondrial biogenesis and function by restoring the balance between glycolysis and OXPHOS, elevating ATP levels, reducing ROS production, and increasing mtDNA copy number and ΔΨm. Furthermore, our data suggest that CAT reduces the severity of TP-induced liver damage, possibly through mechanisms involving the regulation of the SIRT1/HIF-1α pathway. These findings support the potential of CAT as a promising therapeutic candidate for alleviating or treating TP-induced hepatotoxicity.

## Supplementary Material

Supplementary figures and tables.

## Abbreviations

Gck: glucokinase
Pfkl: phosphofructokinase
Pgk1: phosphoglycerate kinase 1
Pklr: pyruvate kinase L/R
Ldha: lactate dehydrogenase A
Cs: citrate synthase
Idh3a: isocitrate dehydrogenase 3 catalytic subunit alpha
Ogdh: oxoglutarate dehydrogenase
Sdhb: succinate dehydrogenase complex iron sulfur subunit B
Fh: fumarate hydratase
Mdh2: malate dehydrogenase 2
cAMP: cyclic adenosine monophosphate
p-FoxO1: phospho- forkhead box O1
Gys2: glycogen synthase 2
Pgm1: phosphoglucomutase 1
Pgm2: phosphoglucomutase 2
Pgm3: phosphoglucomutase 3
Ugp: UDP-glucose pyrophosphorylase
Gyg2: glycogenin 2
Gbe1: 1,4-alpha-glucan branching enzyme 1
Phka2: phosphorylase kinase regulatory subunit alpha 2
Phkb: phosphorylase kinase regulatory subunit beta
Phkg2: phosphorylase kinase catalytic subunit gamma 2
Pck1: phosphoenolpyruvate carboxykinase 1
G6pt: glucose-6-phosphatase catalytic subunit 1
