# Supplementary material for: Catalpol attenuates hepatic glucose metabolism disorder and oxidative stress in triptolide-induced liver injury by regulating the SIRT1/HIF-1α pathway
Source: Int J Biol Sci. 2024 Aug 1;20(10):4077–97. doi: 10.7150/ijbs.97362 (PMC11302874; doi:10.7150/ijbs.97362)
Supplement: Supplementary file 1 — Supplementary figures and tables. [file ijbsv20p4077s1.pdf]

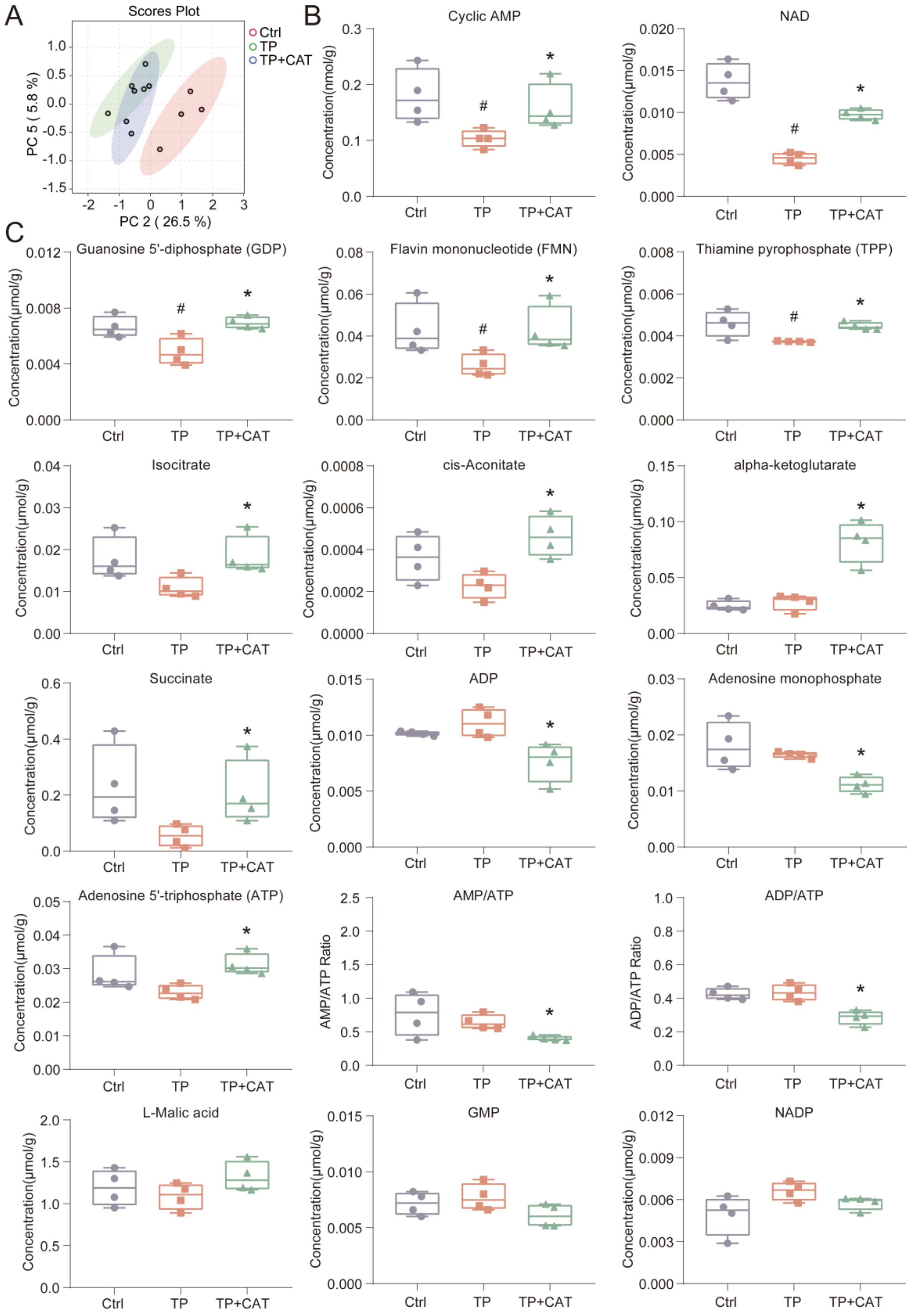

Fig. S1. Catalpol (CAT) improved triptolide (TP) induced energy metabolism disorder in the liver. (A) The PCA score plots for energy metabolites within the liver tissue of the three distinct groups (Ctrl, TP, and TP + CAT) were derived from UPLC-MS/MS assessments. (B-C) Box plots of some hepatic metabolites and their ratios in three groups (Ctrl, TP and TP + CAT groups). Data are expressed as mean ± SD ($n = 4$); $^{\#}P < 0.05$ versus Ctrl, $^{*}P < 0.05$ versus TP.

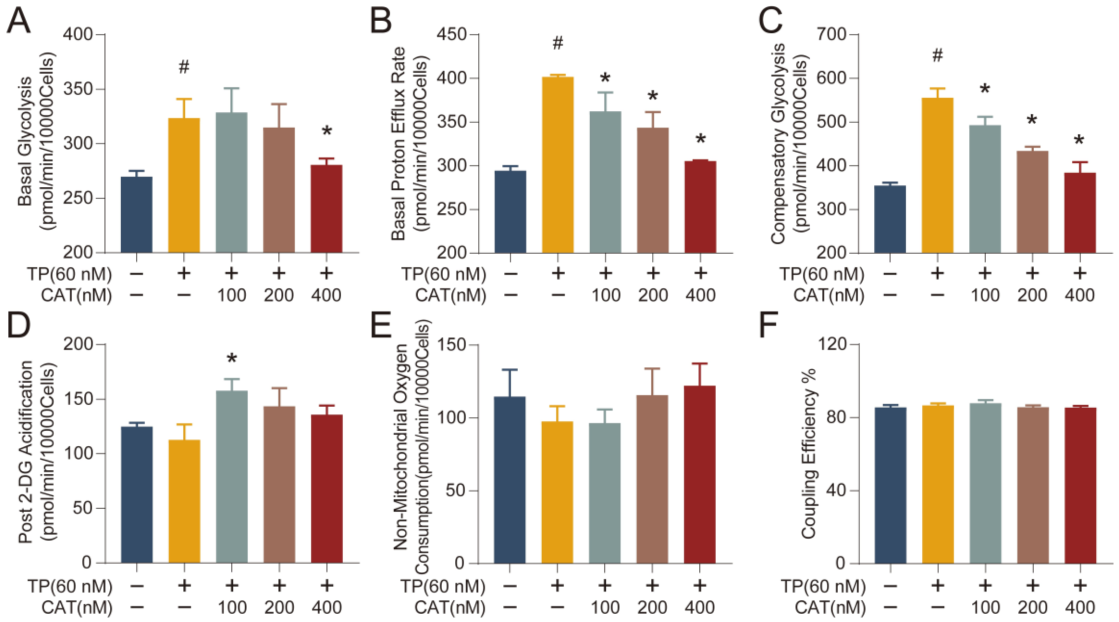

Fig. S2. The beneficial role of catalpol (CAT) was related to regulating the balance between glycolysis and OXPHOS. (A-D) Results from the quantitative analysis of the cellular glycolysis rates: basal glycolysis, basal proton efflux rate, compensatory glycolysis and post 2-DG acidification. (E-F) Results from the quantitative analysis of the cellular respiration parameters: non-mitochondrial oxygen consumption and coupling efficiency %. Data are expressed as mean ± SD (n = 3); $^{\#}P < 0.05$ versus Ctrl, $^{*}P < 0.05$ versus TP.

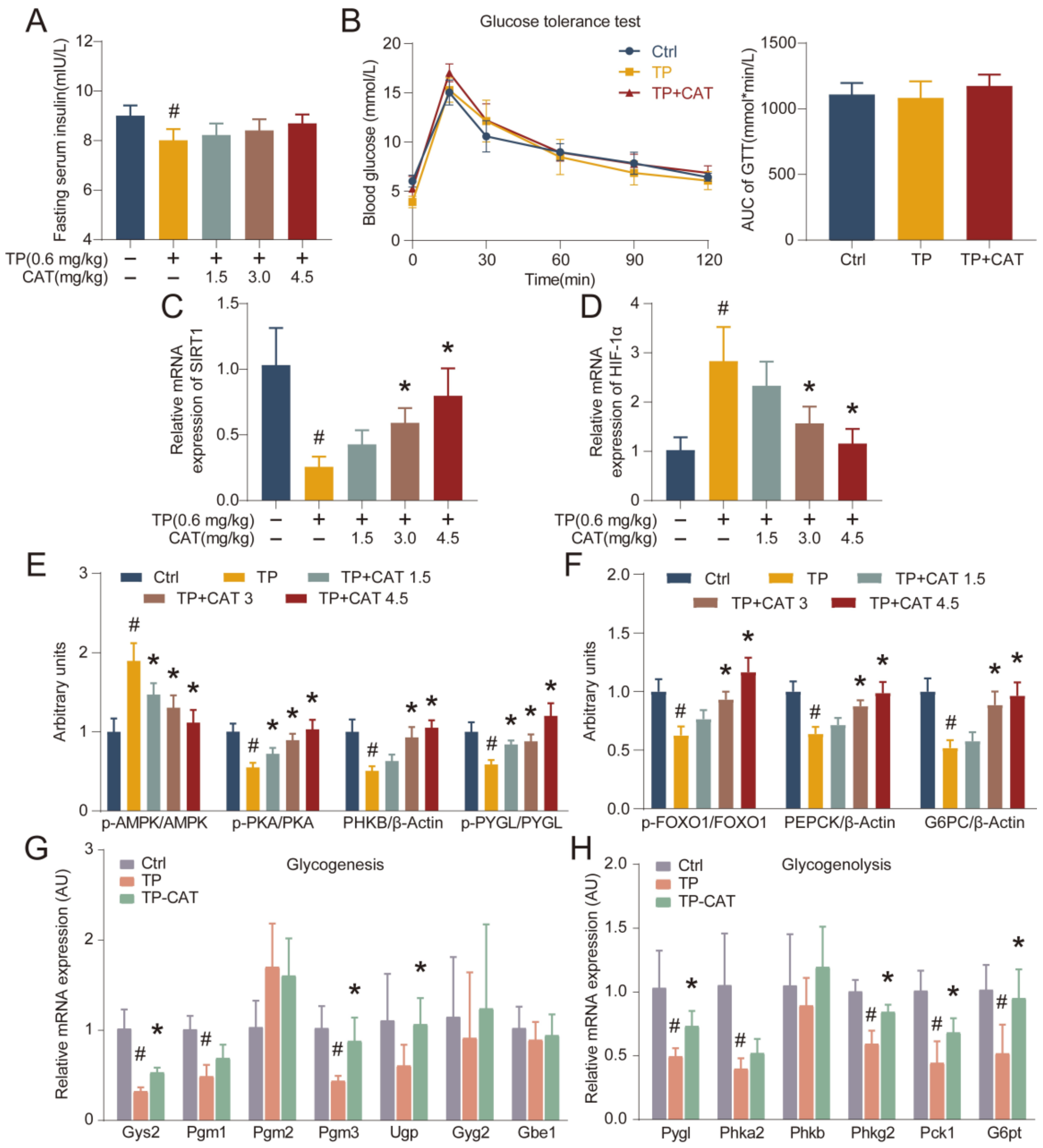

Fig. S3. Catalpol (CAT) alleviated triptolide (TP) induced glycogen metabolism and gluconeogenesis disorders through the SIRT1/HIF-1α pathway. (A) Fasting serum insulin levels. (B) Following a 16-hour fasting period, the mice received an intraperitoneal administration of D-glucose at a dosage of 1 g/kg body weight. Then, blood glucose levels were recorded at specific time intervals. The AUC values for blood glucose were subsequently computed. (C-D) The mRNA expression of SIRT1 and HIF-1α in mouse livers. (E-F) The expression of glycogenolysis and gluconeogenesis related proteins was quantified. (G) Relative expression of glycogenesis-related genes in mouse livers. (H) Relative expression of glycogenolysis-related genes in mouse livers. Data are expressed as mean ± SD (n = 6); $^{\#}P < 0.05$ versus Ctrl, $^{*}P < 0.05$ versus TP.

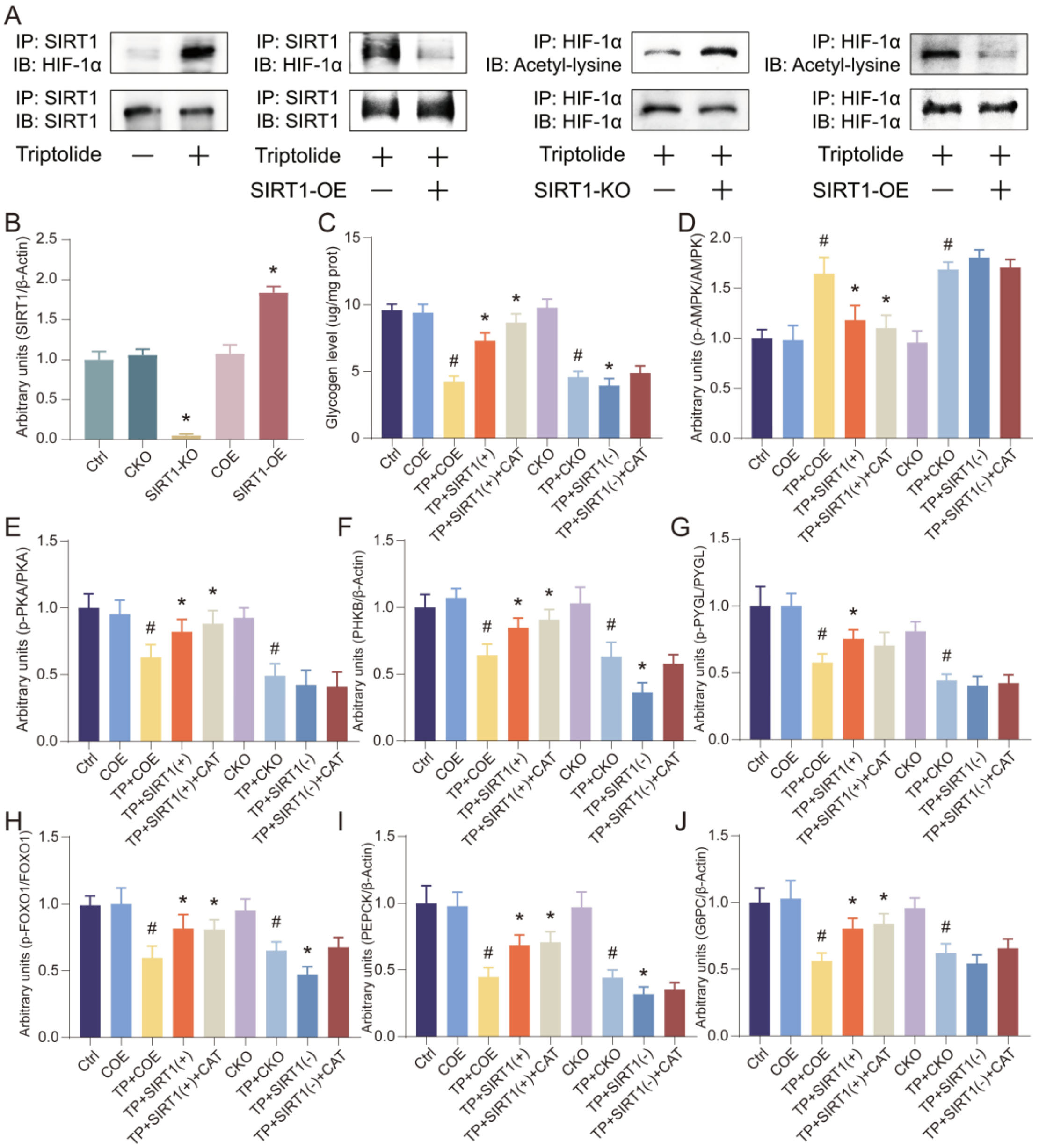

Fig. S4. SIRT1/HIF-1α was the target of catalpol (CAT) and the beneficial effects of CAT were influenced by the overexpression or knockout of SIRT1 *in vitro*. (A) Co-immunoprecipitation was performed using equal protein quantities with either SIRT1 antibody or HIF-1α antibody, followed by immunoblotting procedure using antibodies against SIRT1, HIF-1α or Acetyl-lysine, illustrating the impact of TP, *SIRT1* overexpression (OE) and *SIRT1* knockout (KO). (B) SIRT1 protein expression of transfected cells was quantified. (C) Glycogen levels in transfected cells. (D-J) The expression of glycogenolysis and gluconeogenesis related proteins in transfected cells was quantified. Data are expressed as mean ± SD (n = 3); $^{\#}P < 0.05$ versus Ctrl, COE or CKO, $^{*}P < 0.05$ versus TP, TP + COE or TP + CKO.

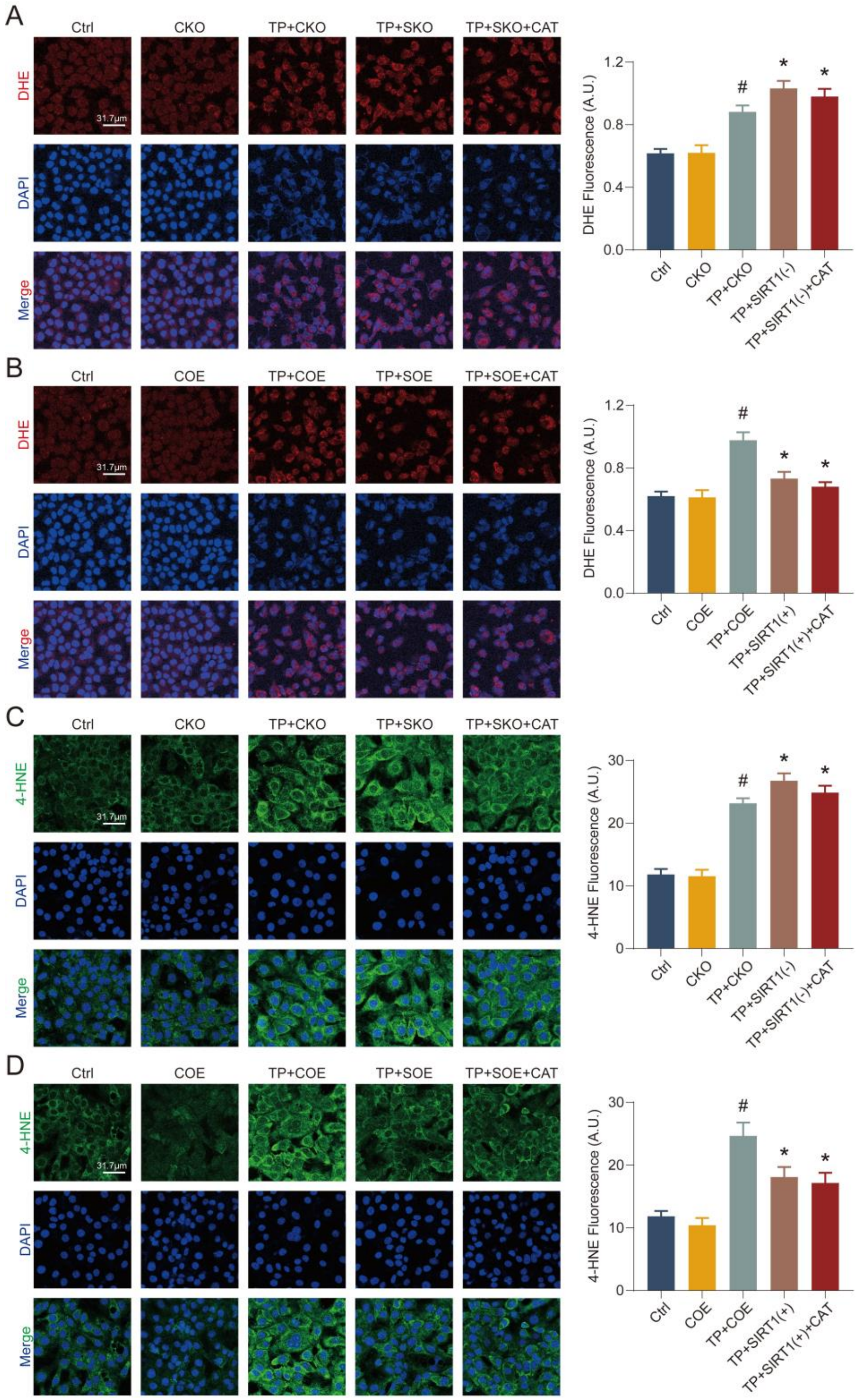

Fig. S5. SIRT1/HIF-1α was the target of catalpol (CAT) and the beneficial effects of catalpol were influenced by the overexpression or knockout of SIRT1 in vitro. (A-B) Representative DHE fluorescence staining of transfected cells and relative quantification of DHE fluorescence images. Scale bar, 31.7 μm. (C-D) Immunofluorescence staining of transfected cells using antibody against 4-HNE and relative quantification of 4-HNE fluorescence images. Scale bar, 31.7 μm. Data are expressed as mean ± SD (n = 3); $^{\#}P < 0.05$ versus Ctrl, COE or CKO, $^{*}P < 0.05$ versus TP, TP + COE or TP + CKO.

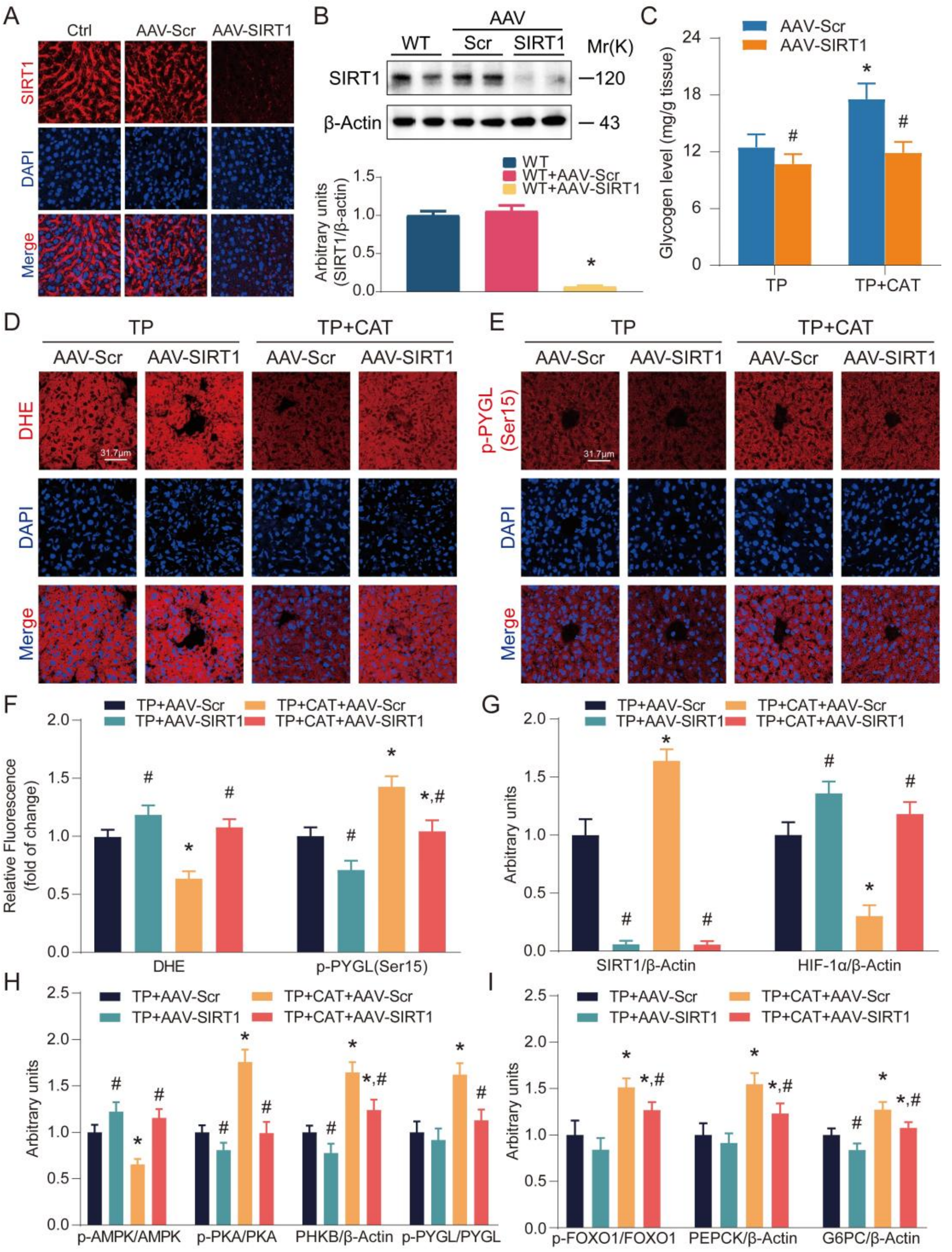

Fig. S6. Liver-specific *SIRT1* knockout aggravated triptolide (TP) induced liver injury and weakened the beneficial effects of catalpol (CAT). (A-B) Validation of liver-specific *SIRT1* knockout in mouse. (C) Glycogen levels. (D) Representative DHE fluorescence staining of liver sections for ROS production. Scale bar, 31.7 μm. (E) Immunofluorescence staining of liver sections using antibodies against p-PYGL(S15). Scale bar, 31.7 μm. (F) Relative quantification of DHE and p-PYGL(S15) fluorescence image. (G) Relative quantification of SIRT1 and HIF-1α protein in mouse livers. (H-I) The expression of glycogenolysis and gluconeogenesis related proteins was quantified. Data are expressed as mean ± SD (n = 3); An "*" indicates significant difference between TP and TP + CAT groups (P < 0.05). A "#" indicates significant difference between AAV-Scr and AAV-SIRT1 in either TP or TP + CAT groups (P < 0.05).

Table S1: Antibodies used in the study

Antibody	Dilution ratio	Supplier	Catalogue Number	RRID
SirT1	1/1000	Cell Signaling Technology	8469	AB_10999470
HIF-1α	1/1000	Cell Signaling Technology	36169	AB_2799095
4-Hydroxynonenal	1/500	R and D Systems	MAB3249	AB_664165
p-AMPKα (Thr172)	1/1000	Cell Signaling Technology	2535	AB_331250
AMPKα1	1/1000	Cell Signaling Technology	2795	AB_560856
p-PKA(T197)	1/1000	ABclonal	AP0557	AB_2771456
PKA	1/5000	ABclonal	A18603	AB_2862360
PHKB	1/1000	Proteintech	13400-1-AP	AB_2237183
p-PYGL (Ser15)	1/1000	Affinity Biosciences	AF3863	AB_2847177
PYGL	1/1000	Affinity Biosciences	DF12134	AB_2844939
p-FoxO1 (S256)	1/1000	ABclonal	AP0172	AB_2771120
FOXO1	1/1000	ABclonal	A13862	AB_2760713
PEPCK/PCK2	1/1000	ABclonal	A4466	AB_2863279
G6PC	1/1000	Proteintech	66860-1-Ig	AB_2882199
Acetyllysine	1/300	PTM BIO	PTM-101	AB_2940830
beta Actin	1/1000	Affinity Biosciences	AF7018	AB_2839420
Tubulin beta	1/1000	Affinity Biosciences	AF7011	AB_2827688
Goat anti-mouse IgG	1/10000	Proteintech	SA00001-1	AB_2722565
Goat anti-rabbit IgG	1/10000	Proteintech	SA00001-2	AB_2722564
CoraLite594 conjugated Goat Anti-Mouse IgG	1/500	Proteintech	SA00013-3	AB_2797133
CoraLite488 conjugated Goat Anti-Rabbit IgG	1/500	Proteintech	SA00013-2	AB_2797132

Table S2: Sequences of primer pairs used for amplification of mRNA by real-time PCR

Gene Description	Primers for qPCR(5’ > 3’)
Gck	F CCGAGATGCTATCAAGAGGAGAGG<br>R CTCACATTGGCGGTCTTCATAGTAG
Pfkl	F GCATCAAGCAGTCAGCCTCAG<br>R CAGTAGCCAGGTAGCCACAGTAG
Pgk1	F GCCAAGTCCGTTGTCCTTATGAG<br>R GCCCAGCAGAGATTTGAGTTCAG
Pklr	F TGTGGTGGCAGTCCGAGATG<br>R ACTTCTTCACGCCTTCATGGTTC
Ldha	F CTGTCACGGCTGGGTCCTG<br>R TCCTTCCACTGCTCCTTGTCTG
Cs	F ACTCATCCTGCCTCGTCCTTG<br>R GCTCCTTAGGTATCAGATTGCTCAG
Idh3a	F ACGGAAGGAGAATACAGTGGAATTG<br>R TTGCTTGCTTCTTCGGTGATGAG
Ogdh	F TGGCTCACTGCTGAAGGAAGG<br>R TGGTGGCGATGGCTGAAGG
Sdhb	F TCTACCGCTGCCACACCATC<br>R GCCAATGCTCGCTTCTCCTTG
Fh	F GTTACCGTTGGAGGCAGCAATG<br>R CTGTGAAGGACACTGAAGCATCTC
Mdh2	F AGGTTCTGCCACTCTGTCCATG<br>R ACACTCAACGACTCCTTCCTTCC
Sirt1	F TGATTGGCACCGATCCTCG<br>R CCACAGCGTCATATCATCCAG
Hif-1α	F TCTCGGCGAAGCAAAGAGTC<br>R AGCCATCTAGGGCTTTCAGATAA
Gys2	F TCAGTACCACCTTCTCCGTCAG<br>R CTCATCCTCATCTTCTTCGTCTTCC
Pgm1	F GGATTGGTCGCCTGGTTATTGG<br>R TCCTGGATTATGGCTGGCTGTC
Pgm2	F AAGACAGTGGTGAGTGGAGAGTG<br>R TGGAGGAAACAGTGCTGGACAG
Pgm3	F CAGACCGCCTTCGTAGTCATTG<br>R AACAGTCACGCCATCTATCACAG
Ugp	F TGAACAACAAGACGCTGGAGAAC<br>R CACGGGCAAGAAACGGGAAC
Gyg2	F AGCACCAGGCGGCATTCC<br>R GCAAAGTGTGTGACCAGGAGAC
Gbe1	F GAACATAAGATGGTGGTTGGAGGAG<br>R TCCGTGGTGATGATAGAGCATAGAG
Pygl	F GCTTGCTGCCTGCTTCCTG<br>R TCTGCCTCTTCTACCTGCCATC

Phka2	F AGAAGACCATATCTACGCCTCTACC<br>R CAATGCTGATGTCCTGTCCACTG
Phkb	F CACAACCGCAACAGGCAGAC<br>R CCAAGGCAAACGCAGGGTAAC
Phkg2	F TCTGTGGTCCGCCGTTGTG<br>R TGTGTGTCTCTCGCCGTGTG
Pck1	F ACTGTTGGCTGGCTCTCACTG<br>R GGGATGGGCACTGTGTCTCTC
G6pt	F AGGTCGTGGCTGGAGTCTTG<br>R CGGAGGCTGGCATTGTAGATG
Cybb	F AGTGCGTGTTGCTCGACAA<br>R GCGGTGTGCAGTGCTATCAT
β-Actin	F GATGGTGGGAATGGGTCAGAAGG<br>R TTGTAGAAGGTGTGGTGCCAGATC
